# Phylogeny-wide conservation and change in developmental expression, cell-type specificity and functional domains of the transcriptional regulators of social amoebas

**DOI:** 10.1186/s12864-019-6239-3

**Published:** 2019-11-21

**Authors:** Gillian Forbes, Zhi-hui Chen, Koryu Kin, Hajara M. Lawal, Christina Schilde, Yoko Yamada, Pauline Schaap

**Affiliations:** School of Life Sciences, University of Dundee, DD15EH, Dundee, UK

**Keywords:** Dictyostelia, Evolution of transcriptional regulation, Evolution of phenotype, Comparative genomics, Comparative transcriptomics, Amoebozoa

## Abstract

**Background:**

Dictyostelid social amoebas self-organize into fruiting bodies, consisting of spores and up to four supporting cell types in the phenotypically most complex taxon group 4. High quality genomes and stage- and cell-type specific transcriptomes are available for representative species of each of the four taxon groups. To understand how evolution of gene regulation in Dictyostelia contributed to evolution of phenotypic complexity, we analysed conservation and change in abundance, functional domain architecture and developmental regulation of their transcription factors (TFs).

**Results:**

We detected 440 sequence-specific TFs across 33 families, of which 68% were upregulated in multicellular development and about half conserved throughout Dictyostelia. Prespore cells expressed two times more TFs than prestalk cells, but stalk cells expressed more TFs than spores, suggesting that gene expression events that define spores occur earlier than those that define stalk cells. Changes in TF developmental expression, but not in TF abundance or functional domains occurred more frequently between group 4 and groups 1–3, than between the more distant branches formed by groups 1 + 2 and 3 + 4.

**Conclusions:**

Phenotypic innovation is correlated with changes in TF regulation, rather than functional domain- or TF acquisition. The function of only 34 TFs is known. Of 12 TFs essential for cell differentiation, 9 are expressed in the cell type for which they are required. The information acquired here on conserved cell type specifity of 120 additional TFs can effectively guide further functional analysis, while observed evolutionary change in TF developmental expression may highlight how genotypic change caused phenotypic innovation.

## Background

Multicellularity enables organisms to specialize their cells for different functions and to organize the specialized cells into a wide array of tissues and organs. Cell-type specialization results from selective gene transcription, which is largely achieved by the binding of sequence-specific transcription factors upstream of the trancription start site in the 5′ intergenic regions of protein coding genes. The regulation of the activity of these factors by intercellular communication and environmental cues is one of the major mechanisms that allow fertilized eggs to develop into functioning adults. The duplication and diversification transcription factor genes and their expression is considered to have been a major mechanism for acquisition of ever-increasing cell-type specialization and organismal complexity in the course of evolution [1].

Dictyostelid social amoebas represent an early type of multicellularity where cells feed as individuals, but come together when starved to form multicellular aggregates. The aggregates transform into migrating slugs and fruiting bodies, which, depending on the species, contain spores and up to four more cell-types [2]. This life cycle evolved from that of the solitary amoebas, which encyst individually when starved. Encystment still occurs in some Dictyostelia, when conditions for aggregation are unfavourable [3].

We aim to understand how the gene regulatory mechanisms that caused cell-type specialization evolved in early multicellular organisms, using the genetically tractable Dictyostelia to investigate this problem. Molecular phylogenies subdivide Dictyostelia into four major and some minor groups [4, 5], with most novel cell types appearing in group 4 [6, 7], which contains the model organism *Dictyostelium discoideum*. Following completion of the *D. discoideum* genome sequence [8], we obtained genome sequences for a representative species in each of the three other taxon groups, which were almost fully assembled by primer walking [9, 10]. Others and ourselves obtained transcriptome data across taxon groups of purified cell types and during developmental progression into fruiting bodies and cysts, both earlier [10–12] and in this work. The high quality genomes and transcriptomes allow us to retrace changes in the abundance, expression profiles, cell type specificity and functional domain architecture of *Dictyostelium* transcriptional factors (TFs) throughout the course of their evolution.

We here present conservation and change in 440 sequence-specific and 42 general TFs of Dictyostelia, highlighting associations between particular TF families and specific developmental roles, taxon group-specific gene amplification and loss, and evolutionary changes in the cell-type specificity and developmental regulation of TFs.

## Results

### Identification and conservation of transcription factor families

The genomes of *D. discoideum* (*Ddis*) and *D. purpureum* (*Dpur*) in group 4, *D. lacteum* (*Dlac*) in group 3, *P. pallidum* (*Ppal*) in group 2 and *D. fasciculatum* (*Dfas*) in group 1 were screened for the presence of members of the 97 known eukaryotic families of sequence–specific transcription factors [13]. Groups 1, 2, 3 and 4 have recently been reclassified as families with the names Cavenderiaceae, Acytosteliaceae, Raperosteliaceae and Dictyosteliaceae, while *Dlac*, *Ppal* and *Dfas* have been renamed to *Tieghemostelium lacteum*, *Heterostelium album* and *Cavenderia fasciculata* [14]. However, this classification was based on the single gene small subunit ribosomal DNA phylogeny [4], which was superseded by more robust multi-gene phylogenies, which only partially support the new classification [5, 15]. We therefore continue to use the older nomenclature here.

In the first round of screening, TFs were retrieved from species proteomes by the Interpro identifier for the functional domain that defines each TF. In the second round, BLASTp or tBLASTn searches were performed on local proteome or genome libraries using signature TF sequences as query. For apparently incomplete orthologous groups, additional BLAST queries were performed with one of the orthologs. Table 1 lists the TF families that were and were not detected in Dictyostelia, with the number of different family members for the former. In total we detected 440 different TF genes, subdivided into 33 families, with 4 families being first identified in Dictyostelia.

**Table 1:** Sequence-specific transcription factors detected in Dictyostelia

Eukaryote sequence-specific transcription factor families			
in Dictyostelia	n	not in Dictyostelia	
AATF	1	AFT	MADF
ARID/BRIGHT	3	Alfin-like	MATα1
AT hook	47	AP2/GBD/EREBP/ERF	MBD
bHLH	4	AP-2/bHSH	mTERF
bZIP	23	APSES/KilA-N	NAC/NAM
C2H2_ZnF	103	B3/VP1/IAA /ARF	PAX
C2HC5_ZnF	1	ABF1_ARS1	PLATZ
CBF/NF-Y	12	BBR/BPC	POU
***Crtf***	2	BES1/BZR1/LAT61	Pros/Prox
***Cud***	6	Brinker	Rap1
CxC	1	CENPB	RFX
E2F/DP	9	CG-1/CAMTA	RHR/RHD
EnY2	1	COE/EBF	Runt
FAR1/FRS	1	Copper-fist	S1FA
Gal4-like	5	Grainyhead/CP2/LSF	SAND/KDWK
GATA	65	CSD	SART-1
***GBF***	6	CSL/LAG1	SBP/SQUAMOSA
GCFC	3	CUT/ONECUT/CDP	Sigma70
HMG	7	DBP/DNC	SMAD/MH1
Hox	30	DM/Doublesex	STK/GeBP-like
HSF	1	EIN3/EIL	T-box
Jmj-C	14	Ets	TCP
Lambda	2	LEAFY/LFY/FLO	TEA/ATTS/TEF
MADS/SRF	6	FKH/Fox	THAP
MIZ	6	GCM	Trihelix/GT
Myb/SANT	56	GCR1	VHR1
Ndt80/PhoG	3	GRAS	Whirly/PBF2
NF-X1_ZnF	4	GTF2I-like	zf-C2HC
Psq	9	HB-PHD/ ZF-HD	zf-C4
STAT	4	IBD	zf-CXXC
***TF2***	1	IRF	zf-Dof
TMF-1	1	LOB/LBD/AS2	zf-HRT
WRKY	3		

Families of eukaryote sequence-specific TFs, retrieved from [13] that were detected in Dictyostelia contrasted to other eukaryote TF families not found in Dictyostelia. The number of different genes (n) detected across *Ddis*, *Dpur*, *Dlac*, *Ppal* and *Dfas* is indicated. Families in italics/bold are unique to Amoebozoa

**Fig. 1:**
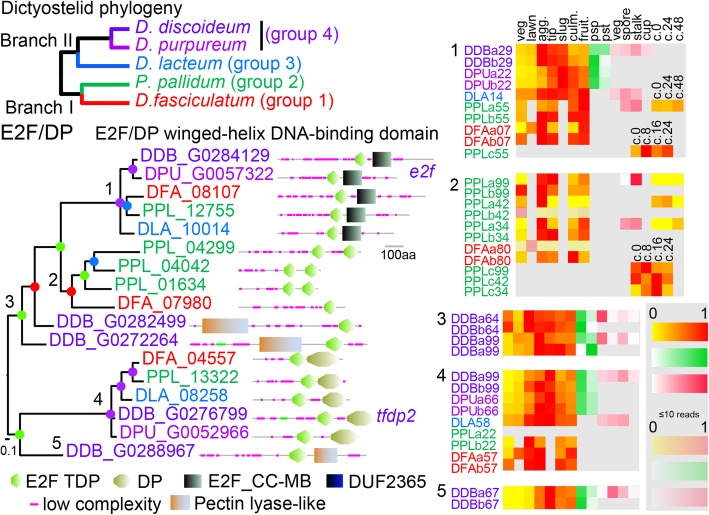
Conservation and change in E2F/DP function and expression across Dictyostelia. Proteins containing E2F/DP winged helix DNA binding domains were identified by their Interpro identifier IPR003316 and BlastP search of five taxon-group representative dictyostelid proteomes. The sequences corresponding to the E2F/DP domains were aligned and a phylogeny was inferred by Bayesian analysis [16], and decorated with the functional domain architecture of the proteins analysed using SMART [17]. Locus tags and gene names are colour coded to reflect the taxon group of the host species, as shown in the dictyostelid phylogeny. Clades of orthologous genes or other groupings are annotated with relative transcript levels, shown as heat maps, at different developmental stages (yellow-red: 0–1 fraction of maximum value), prespore or prestalk cells (white-green: 0–1 fraction of summed reads), or vegetative, spore, stalk and cup cells (white-red: 0–1 fraction of summed reads). Sets with maximally 10 or less reads are shown in wash-out color. The normalized transcript reads were retrieved from published [10–12] or novel RNA sequencing experiments and are all listed in Additional file 2: Table S1. Note that some developmental stages like “lawn” and “slug” are not represented in one or both *Ppal* and *Dfas* time courses. The transcript profiles are preceded by the first three and last two digits of the locus tags, while “a” and “b” represent replicate experiments, except for spore, stalk, cup and vegetative cells where the average of a triplicate experiment was used. Developmental stages: veg.: vegetative; lawn: starving cells, agg.:aggregation; tip: tipped mounds; slug:migrating slugs; culm.:early to mid fruiting bodies; fruit.: completed fruiting bodies, c.0 – c.48: hours into encystation

To understand orthology relationships between family members and map species-specific gene gain and loss, we inferred phylogenetic trees for each family. To assess whether TFs underwent functional change in the course of evolution, the proteins were annotated with their functional domain architectures, which also provided supporting evidence for the orthology of proteins that grouped together. This is for example evident for clades 1 and 4 of the E2F/DP winged helix TFs shown in Fig. 1. To assess whether TFs underwent changes in developmental expression and/or cell type specificity, we used published RNAseq data of *Ddis* and *Dpur* developmental time courses and purified prestalk and prespore cells [11], purified *Ddis* spore-, stalk-, cup- and vegetative cells [12], *Dlac*, *Ppal* and *Dfas* developmental time courses and *Ppal* purified spores and stalk cells [10], as well as unpublished time courses of *Ppal* encystation and *Dlac* purified spore, stalk and vegetative cells. All RNAseq data are comprehensively listed in Additional file 2: Table S1 Because the different species do not complete development at the same time, developmental stages rather than time points were compared between species. For example, Fig. 1 shows that the *e2f* and *tfdp2* genes and their orthologs in group-representative species are all upregulated at aggregation and tend to be more highly expressed in prespore cells in *Ddis* and *Dpur*. Neither gene is consistently upregulated in either of the mature cell types, but the *Ppal e2f* ortholog shows some upregulation in encystation.

**Fig. 2:**
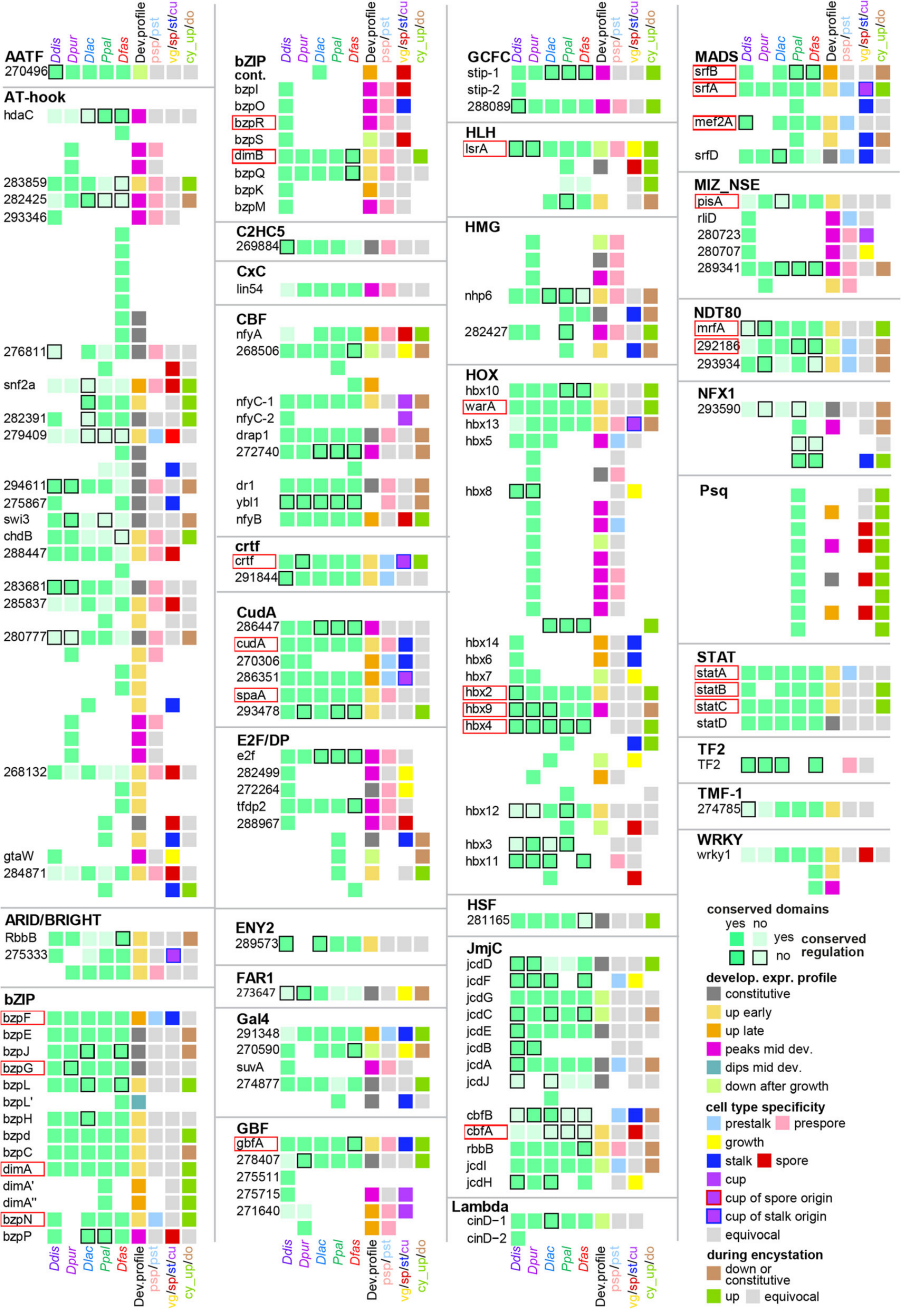
Phylogeny-wide change in sequence-specific TF families with < 50 members. The presence of orthologous TF genes across the *Ddis, Dpur, Dlac, Ppal* and *Dfas* genomes is indicated by green squares below species names, which are shown in a lighter tone or with a black border, when compared to the majority, the functional domains or the developmental regulation, respectively, are not conserved. Where the number of non-conserved features is larger than 3, all differ from each other. The colour coding of the 6th, 7th and 8th square in each row respectively represent the developmental expression profile in the majority of species, the prestalk/prespore specificity when conserved between *Ddis* and *Dpur* slugs, the spore or stalk specificity when conserved between species, the cup and vegetative cell specificity in *Ddis*. The 9th square represents up- or down regulation in encystation of *Ppal*. Cup cells are only present in group 4 and are bordered red or blue when the orthologs in group 2 or 3 show spore- or stalk-specific expression, respectively. Grey reflects lack of specificity or conflicting data between species or replicate experiments and white reflects absence of data. The genes are listed by the *Ddis* gene names or 12 digit Dictybase gene identifiers from which the DDB_G0 prefix was omitted. The names of genes with known biological roles in *Ddis* are bordered in red. The gene identifiers and locus tags for the *Dpur, Dlac, Ppal* and *Dfas* genes are listed in Additional file 1: Table S2 together with all data on which this figure and Fig. 3 and Additional file 1: Figure S18 are based

**Fig. 3:**
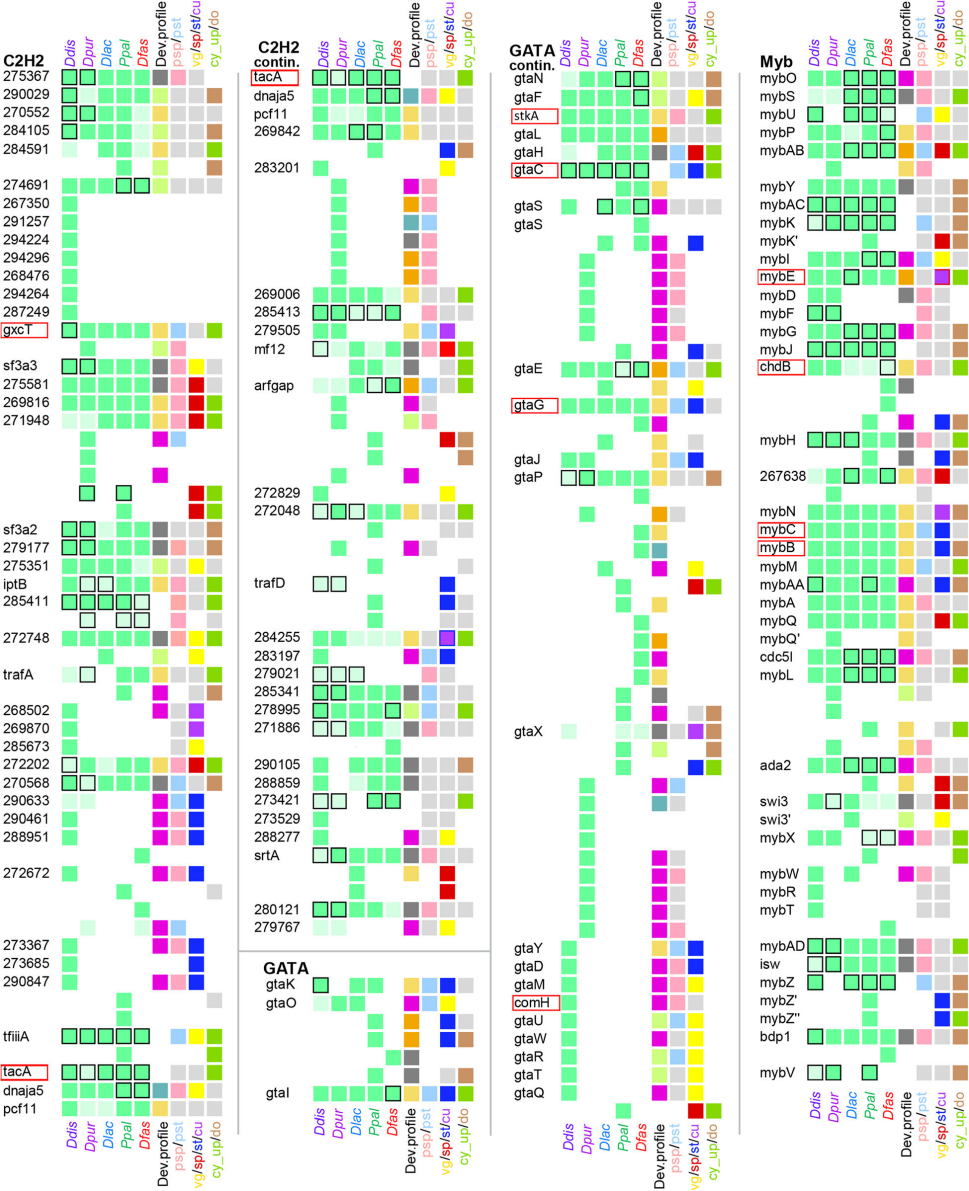
Phylogeny-wide change in sequence-specific TF families with > 50 members. Summary data on conservation of genes and their functional domains, developmental regulation and cell type specificity in TF families with more than 50 members. See the legend to Fig. 2 for explanation of the colour coding of feature states

**Fig. 4:**
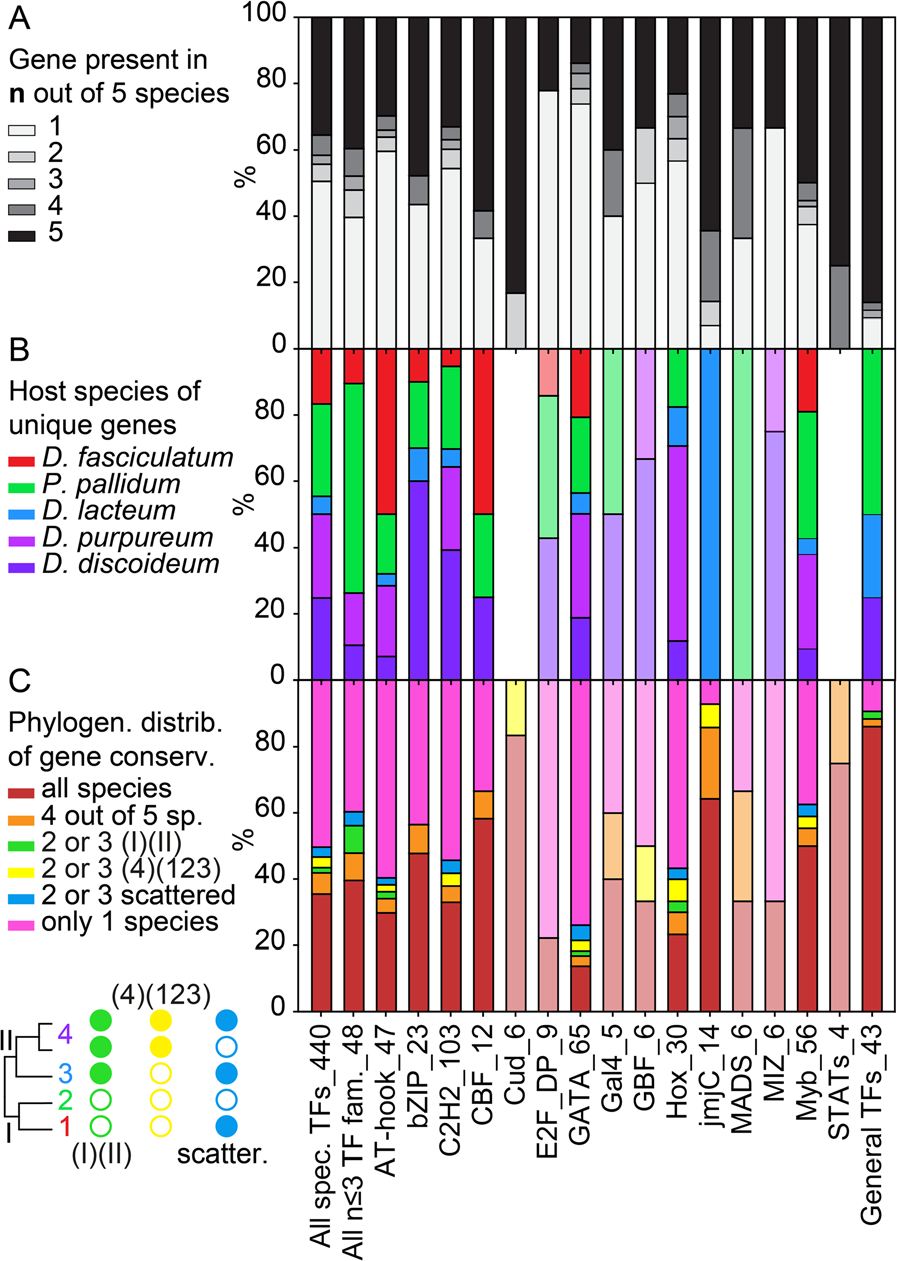
Conservation profiles of TF family members. For each TF family with four or more *D. discoideum* orthologs, for the combined families with three or less members, all combined sequence-specific TFs and all combined general TFs, we calculated the percentage of the different states of the following features: **a**. the total number of orthologs out of five species that were conserved for each gene. **b**. The host species of TFs that were unique. **c**. the phylogenetic distribution of conserved orthologs. The name of each family or grouping and its number of members are shown at the X-axis. For families with less than 10 members, the results are shown in wash-out colour, since they are more likely to be the result of stochastic variation. The figure is based on the data listed in Additional file 3: Table S2 and presented in Figs. 2,3 and Additional file 1: Figure S18

**Fig. 5:**
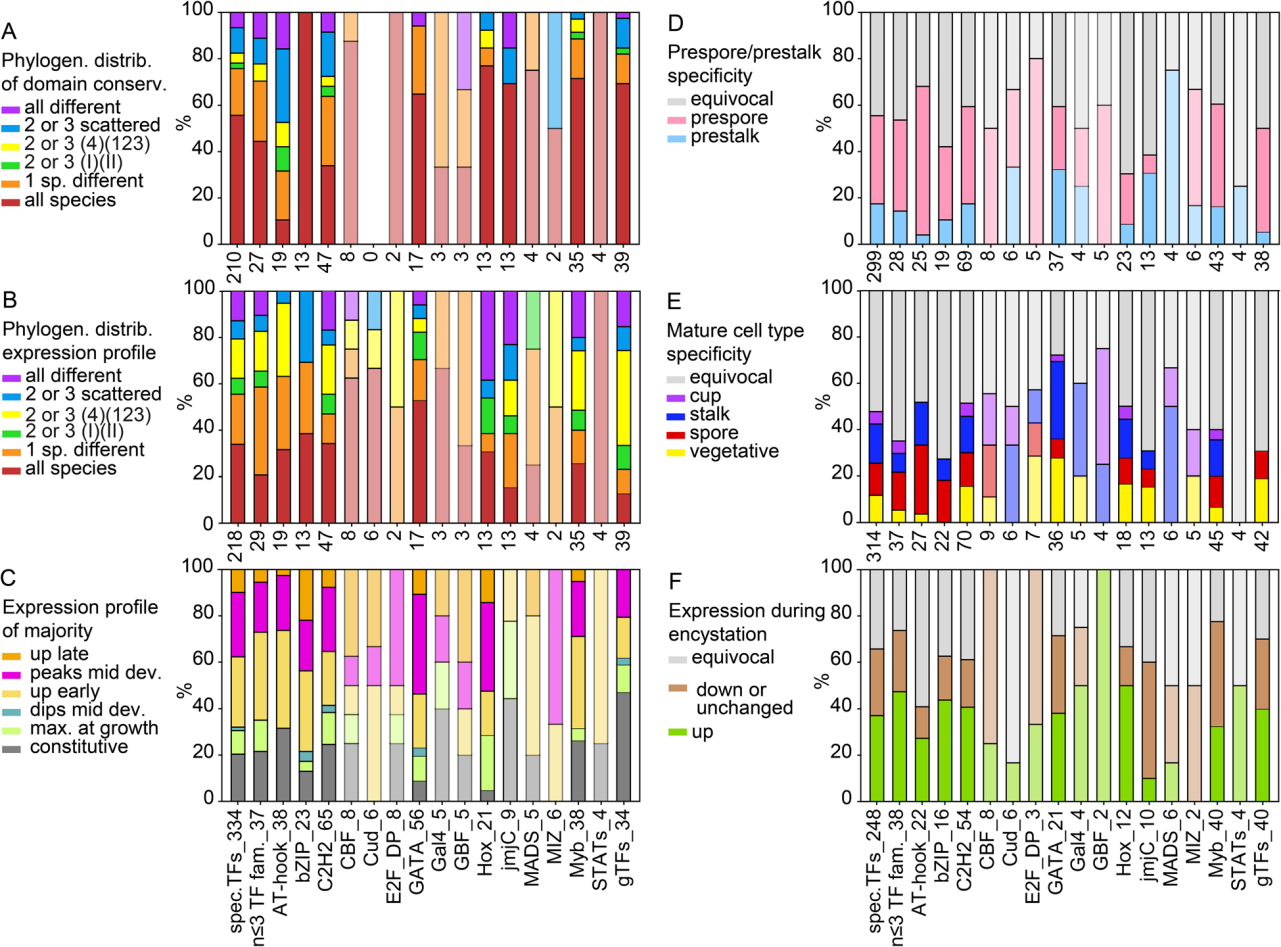
Conservation profiles of functional domains, developmental expression and cell-type specificity. For the same TF groupings as in Fig. 4, we calculated percentages of the different states of the following features: **a, b**. The phylogenetic distribution of respectively the conserved functional domain architecture and developmental expression profiles of conserved orthologs. **c**. The developmental expression profile of the majority of genes within orthologous groups. **d**. The prestalk/prespore preference in *Ddis* and/or *Dpur* slugs. **e**. The cell-type specificity in fruiting bodies of the majority of tested species (*Ddis*, *Dlac* and *Ppal*), compared to vegetative cells. **f**. Expression during encystation in *Ppal*. Note that due to expression data either not being available (**c-f**), or not for at least 2 orthologs, the number of tested orthologs sets (at X-axis) for each TF family or grouping is variable

Similarly annotated phylogenetic trees for all other sequence-specific transcription factor families are shown in Additional file 1: Figure S1 – S16, accompanied by summary descriptions of known roles of the factors within and outwith Dictyostelia. We also searched for orthologs of the general transcription factors (gTFs), which make up and/or associate with the preinitiation complexes that are required for transcription of all genes (Additional file 1: Figure S17). The information on conservation of individual TF genes and their domains, developmental expression and cell-type specificity across Dictyostelia is listed per family in Additional file 3: Table S2 and schematically represented in Figs. 2 and 3 for families with less or more than 50 members, respectively, and in Additional file 1: Figure S18 for the gTFs. For each recorded feature, we also calculated the distribution of the different states of that feature across the individual larger TF families (Figs. 4 and 5).

Overall, 35% of sequence-specific and 86% of gTFs were conserved over all five genomes (Fig. 4a). The *Dpur* genome is most often missing an ortholog, but this is likely an artefact due to it being the only partially assembled draft genome. The large family of GATA TFs shows the most extensive genome-specific gain of individual members. Across sequence-specific TFs, gene amplification occurs about equally frequently in *Ddis*, *Dpur and Ppal,* but is lower in *Dfas* and much reduced in *Dlac* (Fig. 4b), which correlates with and may partially cause the small genome size of *Dlac* (23 Mbp versus ~ 31–35 Mbp for the others [9, 10]).

### Conservation of functional domains and developmental expression

Functional domain architecture is conserved in the greater majority of orthologs (Fig. 5a), except for the AT-hook and C2H2 TFs, where the small domains (12 amino acids for AT-hook, 23 amino acids for C2H2) are often not recognized in some orthologs. Compared to a set of 385 developmentally essential genes [10], the domain architecture of TFs is mostly simple, containing little else than the signature DNA binding domain. There is therefore less opportunity for domain change. More than half of all orthologous sets of TFs show differences in the developmental expression profiles of their member genes. Because change in gene expression may cause individual TF’s to take on novel roles, we were particularly interested in the phylogenetic distribution of such changes. Figure 5b shows that across TF families, developmental expression was most frequently divergent in only one species. In those cases where it was divergent in two or three species, the difference most frequently occurred between group 4 and the other groups and less frequently between the more distantly related branch I and branch II, or scattered across the phylogeny. This is particularly evident in the compiled sets of all sequence-specific TFs, the combined families with three or less members and the general TFs (1st, 2nd and last bars of Fig. 5b) and for the E2F_DP and MIZ TFs. On the other hand, for bZIPs divergent gene regulation occurred only scattered across the phylogeny.

Divergence in functional domain architecture also affects single species most, but is then mostly scattered across the phylogeny (Fig. 5a) and the same is true for conservation of the TF genes themselves (Fig. 4c). This difference between conservation of gene function and conservation of gene expression was also observed for the set of 385 developmentally essential genes, where changes in gene expression were more group 4-specific and changes in functional domains more scattered across the phylogeny [10]. Analysis of 25 phenotypic traits over 99 *Dictyostelium* species showed that the most dramatic changes in phenotype occurred in the last common ancestor to group 4 [6, 7]. The current and earlier analyses of genotypic change indicate that these phenotypic innovations were more likely caused by changes in the regulation of existing genes than by the appearance of novel genes or novel functional domains. The observed limited importance of change in functional domains does however not exclude that more subtle mutations that alter gene function strongly affect phenotypic evolution.

When comparing developmental expression profiles across TF families (Fig. 5c), it is striking that except for the general transcription factors which are mostly constitutively expressed, over 70% of the sequence-specific transcription factors are upregulated after the transition from growth to development, with the small families of Cud and MIZ TFs being exclusively expressed in development. Early upregulation around the aggregate stage or a peak of expression in mid-development are the most dominant expression profiles. Apart from the jmjC TFs, no sequence-specific TFs are predominantly expressed in the vegetative stage.

### Cell-type specificity of transcription factors

To investigate whether families of transcription factors are associated with specific cell fates, we also calculated how families with more than 3 members were percentage-wise expressed in each of the six scored cell types and for *Ppal* in the process of encystation. Across all sequence-specific TFs, 38% was specifically expressed in the prespore cells and 18% in the prestalk cells of group 4 slugs, and this difference was even more extreme for the general TFs with 45 and 5% expression in prespore and prestalk cells respectively (Fig. 5d). Only the JmjC and GATA families contained more members with prestalk than prespore expression, while no MADS or STAT TFs were specifically expressed in prespore cells and no E2F_DP, CBF or GBF TFs in prestalk cells.

In the fruiting body stage, this cell fate specificity was almost reversed for the sequence-specific TFs, of which 14% were expressed in spores and 17% in stalk cells (Fig. 5e). Another 5% of TFs were expressed in cup cells, a population that is derived from prestalk cells [12, 18, 19]. This suggests that most genes that define the spore phenotype are already expressed in the slug stage, but that those that define the stalk and cup phenotypes are only expressed late in fruiting body formation. Here there was also evidence for more cell-type preference of TF families, with bZIP and AT-hook TFs favouring expression in spores and the GATAs, Hox TFs and members of the small families of Gal4, MADS and Cud TFs favouring expression in stalk cells. CBFs, GBFs and MIZ TFs favour expression in cup cells. For the MADS TFs, their stalk and cup preference is consistent with their prestalk preference, but for the GBFs it is the reverse of their prespore preference.

As was also evident from the developmental profiles (Fig. 5c), many more sequence-specific TFs are specifically expressed during development into fruiting bodies than in the vegetative stage, but this not the case for the general TFs, which as expected are more constitutively expressed. Finally, in *Ppal*, where in addition to multicellular development, starving amoebas can also individually encyst, over 30% of members of all families are upregulated during the encystation process.

### Predicted roles for TFs from cell-type specificity and developmental profiles

**Fig. 6:**
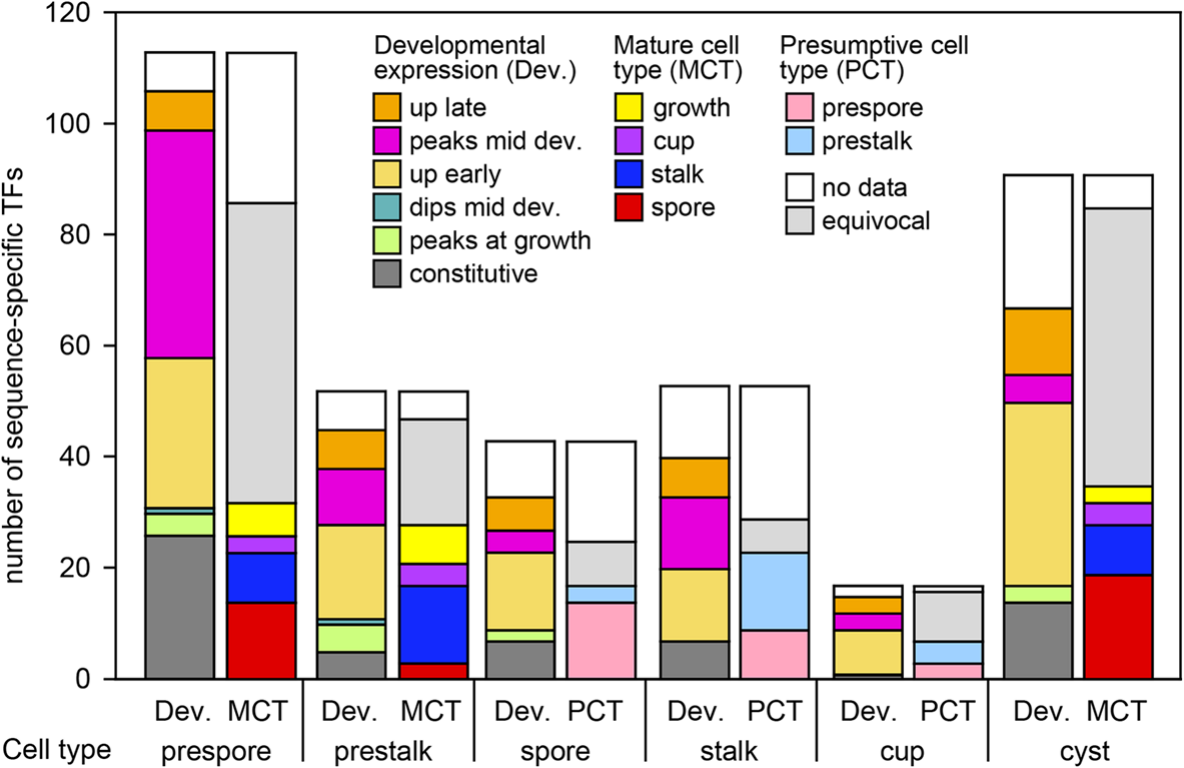
Expression subsets of cell-type specific transcription factors. The sets of prestalk, prespore, spore, stalk, cup and cyst-specific TFs were subdivided into subsets according to the developmental stages at which they were expressed (Dev. bars). Prespore, prestalk and cysts TFs were subdivided in sets according to the mature cell types – spore, stalk, cup, feeding – in which they were expressed (MCT bars), while spore, stalk and cup-specific TFs were subdivided into sets according to expression in prestalk and prespore cells (PCT bars). The total numbers of TFs in each subset are shown. The identities of all TFs in the subsets are listed in Additional file 4: Table S3

Information on stage- and cell-type specificity provides a clue on the possible developmental role of individual TFs and we therefore subdivided individual transcription factors into sets according to the cell-type and stage at which they are expressed. The sets with different cell-type specificity are listed in Table 2 and sets sorted with respect to similar developmental stage of expression or different combinations of stage- and cell type specificity are listed in Additional file 4: Table S3. For an overview that combines data on TF expression in mature (MCT) and presumptive (PCT) cell types and stage of expression, we subdivided all cell type specific TFs into subsets according to their developmental expression profile and presumptive or mature cell fate. Figure 6 shows that prepore-specific TFs mostly show peak expression in mid development or are upregulated early, while out of 113 prespore-specific TFs, only 14 are also spore-specific and 9 become stalk-specific. The number of prestalk-specific TFs is at 52 less than half that of the prespore TFs and most prestalk TFs are upregulated early. 14 prestalk TFs are also stalk-specific, while 3 become spore-specific. Of the 17 cup-specific TFs, 4 were enriched in prestalk cells and 3 in prespore cells. Of the 91 TFs that are upregulated in *Ppal* cysts, 50 are also upregulated in multicellular development. 19 cyst-upregulated TFs are also expressed in mature spores and 9 in stalk cells. Like cysts, spores and stalk cells are surrounded by cellulosic walls. Apparently encystation shares many TFs with multicellular development, with both processes adapting cells to starvation and their metabolism towards cell wall biosynthesis.

**Table 2:** Cell-type specific transcription factors

Prespore-specific		Prespore-contin.		Spore-specific		Prestalk-specific		Stalk-specific	
Family	Gene	Family	Gene	Family	Gene	Family	Gene	Family	Gene
**AThook**	283859	**Cud**	**cudA**	**AThook**	snf2a	**AThook**	279409	**AThook**	275867
	282425		**spaA**		279409	**bZIP**	**bzpF**	**bZIP**	**bzpF**
	293346	**CxC**	lin54		288447		**bzpN**		bzpO
	276811	**E2DP**	e2f		285837	**C2H2**	270568	**C2H2**	290633
	snf2a		272264		268132		290633		290461
	294611		tfdp2		284871		279505		288951
	288447		288967	**bZIP**	bzpP		283197		272672
	283681	**Gal4**	suvA		bzpI		285341		273367
	285837	**GATA**	**stkA**		bzpS		278995		273685
	280777		gtaD	**C2H2**	275581	**Crtf**	**crtf**		290847
	268132		gtaM		269816		291844		trafD
	284871		comH		271948	**Cud**	270306		283197
**bZIP**	bzpI	**GBF**	**gbfA**		272202		286351	**CudA**	**cudA**
	bzpO		271640		mf12	**Gal4**	291348		270306
	**bzpR**	**GCGF**	288089	**CBF**	nfyA	**GATA**	gtaK	**Gal4**	291348
	**dimB**	**HLH**	**lsrA**		nfyB		gtaO	**GATA**	gtaK
	bzpQ	**HMG**	nhp6	**E2DP**	288967		gtaI		gtaI
	bzpM		282427	**GATA**	gtaH		gtaH		**gtaC**
**C2H2**	275367	**Hox**	hbx13	**Hox**	hbx11		**gtaC**		**gtaG**
	sf3a3		hbx5	**JmjC**	**cbfA**		gtaE		gtaJ
	275581		hbx11	**myb**	mybAB		**gtaG**		gtaY
	269816	**JmjC**	rbbB		mybK		gtaJ		gtaD
	271948	**MIZ**	280723		267638		gtaY	**GBF**	**gbfA**
	279177		289341		mybQ		gtaU	**Hox**	hbx14
	280121	**myb**	mybO		swi3		gtaR		hbx6
	iptB		mybS	**WRKY**	wrky1	**Hox**	hbx5	**JmjC**	cbfB
	285411		mybP	**Cup-specific**		**JmjC**	jcdF	**MADS**	srfD
	272748		mybD	Family	Gene		jcdA	**myb**	mybH
	272202		mybF	**ARID**	275333		cbfB		**mybC**
	290461		mybG	**C2H2**	268502		jcdI		**mybB**
	288951		**chdB**		269870	**MADS**	**srfA**		mybAA
	272672		mybH		279505		**mef2A**		mybZ
	273367		267638		284255		srfD		
	290847		**mybA**	**CBF**	nfyC-1	**MIZ**	rliD		
	dnaja5		cdc5l		nfyC-2	**Myb**	mybU		
	285413		ada2	**Crtf**	crtf		mybAB		
	mf12		mybX	**Cud**	275333		mybK		
	271886		mybW	**GATA**	268502		mybI		
	srtA		mybAD	**GBF**	269870		**mybC**		
	280121		isw		279505		mybM		
**C2HC5**	269884		bdp1	**Hox**	284255		mybZ		
**CBF**	nfyA	**TF2**	tf2	**MADS**	**srfA**	**NDT80**	292186		
	drap1			**MIZ**	280723		293934		
	dr1			**myb**	**mybE**	**STAT**	statA		
	ybl1				mybN				

All TFs that are specifically expressed in the prestalk or prespore cells of slugs or in the spore, stalk and cup cells of fruiting bodies in the majority of species are listed. The second column contains either the gene name for annotated genes or the Dictybase 12 digit gene identifier minus the DDB_G0 prefix. Gene names/IDs are shown in bold when the biological role of the gene in *Ddis* is known

Lastly, we explored the extent to which cell type specificity predicts TF function. Of the 254 TFs detected in *Ddis*, there is only functional information from gene knock-outs and knock-down studies for 34 TF genes. Deletion of 12 TFs causes specific defects in, or lack of, terminally differentiated cell types and 9 of these TFs are only expressed in the cell type that is lost upon knock-out (Additional file 5: Table S4). Deletion of 9 TFs causes alterations in the proportion of prespore and prestalk cells. Of this set only 2 TFs are specific to the diminished cell-type and 1 TF is specific to the increased cell type. The remaining 6 TFs are not cell-type enriched. This suggest that cell-type specificity of TFs predicts their role in ultimate cell fate well, but that cell type proportioning is subject to more subtle cross-regulation. Also, logically, a TF that instigates a presumptive cell fate has to be present before that fate is assigned.

## Discussion

Across five genomes that represent the four major groups of Dictyostelia, around 440 different sequence-specific TFs across 33 TF families were detected. Due to genome- and species-specific gene amplification, this is about twice the number of TFs present in individual genomes. For instance, we detected 254 TFs in *Ddis* (as opposed to 106 in the initial genome annotation [8]), of which a core set of 181 TFs is conserved across at least three other genomes.

The large family of GATA TFs is subject to extensive single gene amplification and the number of conserved genes in this family is therefore low. On the other hand, members of the almost equally large family of Myb TFs are mostly conserved. Nine members of the Pipsqueak family are unique to one genome (*Ppal*) and are all strongly upregulated in encystation. Gene amplification occurred about equally across four genomes, but was much lower in the *Dlac* genome, which is also 1/3rd smaller than the other four.

Changes in developmental expression profiles of conserved TFs occurred more frequently between group 4 and groups 1–3, than between the more distantly related branches I and II. This correlates with phenotypic change, which is also most pronounced between group 4 and the other three groups [6, 7]. Since group 4 has neither more novel TFs nor more different functional domains in its TFs, this suggests that altered expression of existing TFs plays an important role in phenotypic innovation.

There are marked differences between TF families in developmental expression with e.g. 78% of bZIPs being developmentally up-regulated and 77% of jmjC TFs being constitutively expressed or developmentally down-regulated. Not surprisingly, most (65%) of the general TFs are constitutively expressed or down-regulated after growth, but across all sequence-specific TFs, 68% are developmentally up-regulated. This suggests that most of the Dictyostelid sequence-specific transcriptional machinery serves the developmental programme, with a relatively low number of TFs left to adapt cells to environmental challenges in the growth stage.

The prespore cells in slugs express over two times more TFs than the prestalk cells, with particularly many AT-hook, CBF, E2F-DP, GBF and general TFs being prespore-specific. However, this changes in the fruiting body stage, when the stalk cells express somewhat more TFs, with some smaller families like the CudA-like, Gal4-like, GbfA-like and MADS TFs being solely expressed in cells of the stalk and cup. Strikingly, TFs that are essential for spore formation, such a cudA, spaA and stkA [20–22] are expressed in prespore, but not spore cells, as if upon sporulation their task is finished. This pattern is similar across all prespore-specifc TFs, of which only 12% persists into the spores. For the prestalk-specific TFs, 34% remain expressed in the stalk and cup. This temporal disparity in cell type specific gene expression likely reflects the different ontogenies of the mature cell types. The prespore cells start prefabrication of the spore wall in Golgi-derived vesicles after aggregation. The vesicles fuse with the plasmamembrane during spore maturation, thus rapidly completing the cell wall [23]. In contrast, stalk cells start cell wall synthesis gradually from the tip at the onset of fruiting body formation, while most cup genes are only expressed once the fruiting body is fully formed [12].

About 34 of the 254 TF genes of *Ddis* have been deleted, resulting in specific loss of or severely defective mature cell types for 12 TFs. For 9 out of 12 cases, the TF was in normal development expressed in the affected cell type and all 12 TFs were conserved throughout Dictyostelia. This implies that bioinformatics-based evidence on cell-type specificity and gene conservation is likely a useful tool for guiding discovery of the function of many of the remaining 220 TF genes.

## Conclusions

Dictyostelia jointly contain 440 different sequence-specific TFs, which are subdivided across 33 families, of which four are thus far unique to Amoebozoa.

Only 32% of sequence-specific TFs are expressed constitutively or during growth, while the rest is developmentally up-regulated, indicating that most of transcriptional machinery serves the multicellular phase of the life cycle.

Changes in developmental expression of TFs, but not in TF functional domains or TF gene gain or loss, are correlated with major changes in phenotype across Dictyostelia, suggesting that altered expression of TFs is a major driver of phenotypic change.

The study presents detailed information on cell-type specificity of TFs, which correlates with an essential role in cell differentiation for 9 out of 12 TFs with known functions. This makes the current analysis an effective tool for gene function discovery.

## Methods

### Sequence retrieval and phylogeny reconstruction

TF protein sequences were firstly retrieved from the *Ddis*, *Dlac*, *Ppal* and *Dfas* genomes using the Interpro (https://www.ebi.ac.uk/interpro/) domain identifiers of all known TF families as query in the “advanced search” option of the social amoeba comparative genome browser SACGB (http://sacgb.fli-leibniz.de/cgi/index.pl). For *Dpur* a similar query was performed in the Pubmed “protein” option (https://www.ncbi.nlm.nih.gov/pubmed) with the combined query “*Dictyostelium purpureum* and [Interpro domain identifier]”. Next, a BLAST library was prepared in CLC-workbench v8.0 (https://www.qiagenbioinformatics.com) from the combined *Ddis*, *Dpur*, *Dlac*, *Ppal* and *Dfas* proteomes, downloaded from Dictybase (http://dictybase.org/) and SACGB, which was queried with the protein sequences of representative functional domains of each TF family.

The domain architectures all detected proteins were analysed using SMART [17], with the visual display of the architecture saved as an .svg file. The domain coordinates were used to isolate the sequences corresponding to the TF functional domains. These sequences were subsequently aligned using Clustal Omega [24] with 5 combined iterations. When functional domain sequences were short, a stretch of 20 amino-acids flanking the domain on either side was included in the alignment. Phylogenies were constructed using RAxML in Topali v2.5 [25] or MrBayes v3.2.6 [16], with the latter run for 10^6^ generations, using a mixed amino acid model with rate variation between sites estimated by a gamma distribution. When otherwise conserved genes appeared to be absent from species, their proteomes or genomes were queried once more by BLASTp or tBLASTn, respectively, using the orthologous sequence as bait. Phylogenetic trees were then reconstructed, including the novel sequences. Trees were rooted at midpoint using FigTree v1.3.1. and saved as .svg files. The tree .svg file was combined with the domain architecture .svg files for each protein in Adobe Illustrator CS5.

### RNA sequencing and analysis

To obtain total RNA for *Dlac* stalk, spore and vegetative cells, amoebas were co-cultured with *Klebsiella aerogenes* on lactose-peptone agar. For vegetative cells, cells were harvested before bacteria started to clear. For stalk and spore cells, cells were harvested, freed from bacteria and incubated for 24 h on non-nutrient agar until fruiting bodies had formed. Spores were separated from stalks and RNA was isolated from the three cell types as described previously [12]. The qualities of the RNAs isolated in three independent experiments were assessed with TapeStation (Agilent) to be good (RIN > 7.5) and cDNA libraries were prepared using the Truseq Stranded mRNA Library Prep Kit (Illumina) with Low Sample Protocol. 75-bp paired end reads were sequenced with Illumina NextSeq 500 at the Tayside Centre for Genomic Analysis in two independent runs. The qualities of the RNA-Seq reads were inspected with FastQC [26]. The RNA-Seq reads were then mapped to the previously assembled transcriptome of *D. lacteum* [27] using RSEM [28] with the bowtie2 aligner and with the read start position distribution (RSPD) estimation option. The read counts were normalized to Transcripts Per Million (TPM) [29] with RSEM.

To monitor gene expression during *Ppal encystation, Ppal* PN500 was co-cultured with *K. aerogenes* on LP agar. Cells were freed from bacteria and incubated at 2.5 × 10^6^ cells/ml in 250 mM sorbitol in 20 mM K-phosphate to induce encystation [30]. Total RNA was extracted with an RNAeasy Midi Kit (Qiagen), directly after harvest (t = 0 h) and after 8, 16 and 24 h of incubation at 22 °C, at which point 80% of cells had encysted. Library construction, sequencing and sequence quality control and mapping of transcripts to the *Ppal* genome [9] were performed by Eurofins Genomics (https://www.eurofinsgenomics.eu/). Paired-end Illumina sequencing was performed on the Hi-seq2000 platform using the TruSeq (TM) SBS v5 sequencing kit. A total of 177,292,620 reads containing 8.8 Mb were obtained. The reads were mapped to the *Ppal* genome, using BWA 0.5.8c software (http://bio-bwa.sourceforge.net). The read counts were then normalized to reads per kilobase per million mapped reads (RPKM).

### Comparative transcriptomics

For comparative analysis of developmental expression and cell type specificity of TF genes across the Dictyostelid phylogeny, normalized read counts from published and purpose-sequenced gene expression studies were combined into a single spreadsheet (Additional file 2: Table S1). The data include i. replicate developmental profiles for *Ddis* and *Dpur* obtained by Illumina sequencing, combined with RNAseq data of purified prestalk and prespore cells of migrating slugs [11], ii. Averaged read counts of three RNAseq experiments comparing purified spore-, stalk- and cup cells from mature *Ddis* fruiting bodies and vegetative cells [12], iii. Averaged read counts of three RNAseq experiments comparing purified spore- and stalk cup cells from *Dlac* fruiting bodies and vegetative cells. iv. A single developmental profile for *Dlac* and replicate developmental profiles for *Ppal* and *Dfas* [10], combined for *Ppal* with RNAseq data of purified stalk and spore cells and 24 and 48 h time points of encystation, vi. A separate 24 h time course of *Ppal* encystation. The developmental profiles are aligned between species with respect to developmental stage, rather than developmental time because species do not develop at the same rate. For each set of orthologous genes, or groups of amplified genes, the normalized read counts for each of the features listed above were transferred to Excel files and recalculated as fraction of the maximum read count for developmental profiles and as fraction of the sum of counts for cell-type specificity data. The conditional formatting option in Excel was used to generate heat maps, which were matched up with the phylogenetic trees in Adobe Illustrator.

## Supplementary information

**Additional file 1: Figure S1-S18.** Annotated phylogenetic trees of transcription factor families.

**Additional file 2: Table S1.** Gene expression profiles. Normalized transcript read counts of developmental time series and purified cell types from five *Dictyostelium* species.

**Additional file 3: Table S2.** Transcription factor conservation. Conservation and change in the presence, developmental expression and functional domain architecture in transcription factors across five Dictyostelid genomes.

**Additional file 4: Table S3.** Transcription factors grouped by cell-type and stage specificity. The data of Additional file 3: Table S2 were compiled and sorted to generate sets that were expressed in the same cell type or at the same developmental stage, or that shared a combination of the same stage and cell type specificity.

**Additional file 5: Table S4.** Transcription factors with known knock-out phenotypes. Knock-out phenotypes of transcription factors combined with their cell-type- and stage specificity of expression.

## Data Availability

The raw RNA-Seq data of *Dlac* cell types and *Ppal* encystation time series have been submitted to Arrayexpress https://www.ebi.ac.uk/arrayexpress/experiments/E-MTAB-7824 under accession number E-MTAB-7824. All other data generated or analysed during this study are included in the published article and its supplementary information files.

## Abbreviations

BLAST: Basic Local Alignment Search Tool
Ddis: Dictyostelium discoideum
Dfas: Dictyostelium fasciculatum
Dlac: Dictyostelium lacteum
Dpur: Dictyostelium purpureum
Ppal: Polyspondylium pallidum
SMART: Simple Modular Architecture Research Tool
TF: Transcription factor
