# Supplementary material for: Phylogeny-wide conservation and change in developmental expression, cell-type specificity and functional domains of the transcriptional regulators of social amoebas
Source: BMC Genomics. 2019 Nov 21;20:890. doi: 10.1186/s12864-019-6239-3 (PMC6873476; doi:10.1186/s12864-019-6239-3)
Supplement: Supplementary file 1 — Additional file 1: Figure S1-S18. Annotated phylogenetic trees of transcription factor families. [file 12864_2019_6239_MOESM1_ESM.pdf]

ADDITIONAL FILE 1 - Supplementary figures S1-S18

Conservation of domain architecture, developmental regulation and cell-type specificity of *Dictyostelid* transcription factors

Contents

Figure S1. AATF and ARID/BRIGHT 2
Figure S2. AT-hook DNA binding domain 3
Figure S3. bZIP 5
Figure S4A. C2H2 ZnF set 1 7
Figure S4B. C2H2 ZnF set 2 8
Figure S4C. C2H2 ZnF set 3, C2H5 and CXC. 9
Figure S5. CBF/NF-Y/Archaeal Histone 11
Figure S6. Crtf and cudA-like transcription factors 12
Figure S7. E2F/DP, EnY2, FAR1 and Gal4-like transcription factors 13
Figure S8A. GATA ZnF set 1 14
Figure S8B. GATA ZnF set 2 15
Figure S9. G-box binding factor 16
Figure S10. GCFC, HLH, HMG and HSF 17
Figure S11. Homeo domain 18
Figure S12. Jumonji C 19
Figure S13. Lambda, MADS and MIZ/NSE 20
Figure S14A. Myb DNA binding proteins set 1 21
Figure S14B. Myb DNA binding proteins set 2 22
Figure S15. NDT80, NFX1 and Pipsqueak 24
Figure S16. STAT, TF2, TMF-1 and WRKY 25
Figure S17A. General transcription factors set 1 26
Figure S17B. General transcription factors set 2 27
Figure S18. Phylogeny-wide change in general transcription factors 28
Supplementary references 29

Figure S1. AATF and ARID/BRIGHT

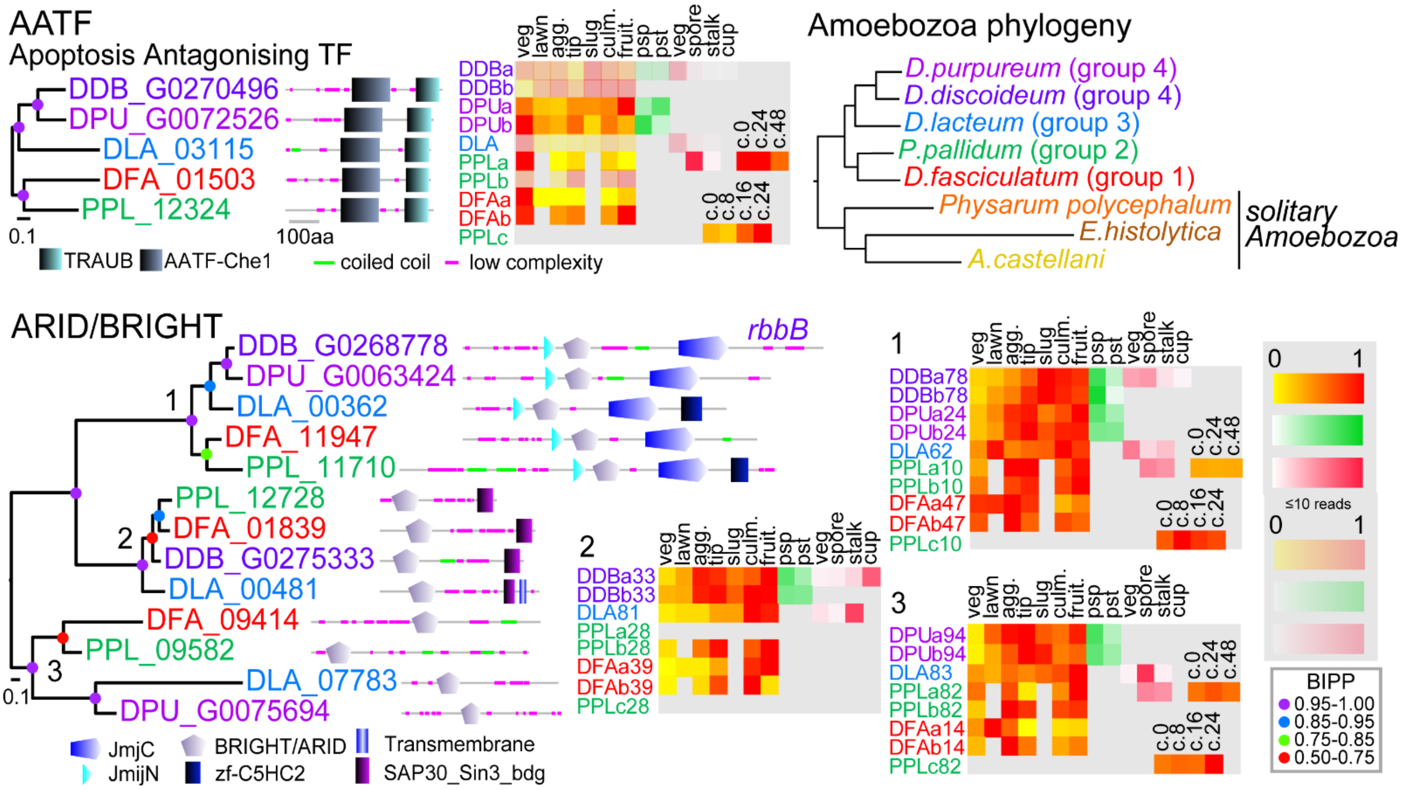

Figure S1. AATF and ARID/BRIGHT transcription factors
Apoptosis Antagonising Transcription Factor genes were retrieved from Dictyostelid genomes with the Interpro IPR012617 identifier and BlastP search with *Ddis* AATF. ARID/BRIGHT genes similarly were retrieved using the Interpro identifier IPR001606 and by BlastP search. The sequences corresponding to functional domains were aligned using Clustal Omega with five iterations (Sievers and Higgins 2014) and a phylogeny was constructed by Bayesian analysis (Ronquist and Huelsenbeck 2003) and decorated with the functional domain architecture of the proteins using SMART (Schultz et al. 1998). Gene IDs and locus tags are colour coded to reflect the taxon group of the host species, as indicated in the Amoebozoa phylogeny. *Ddis* sequences show names for known genes, which are framed in red the biological function of the gene in *Ddis* is known. Clades of orthologous genes or other groupings are annotated with relative transcript levels at specific developmental stages or specific cell types, shown as heat maps that represent fraction of maximum transcript number for the developmental profiles and fraction of the summed number for the cell types. The normalized transcript numbers were retrieved from published RNA sequencing experiments (Gloeckner et al. 2016; Kin et al. 2018; Parikh et al. 2010b) and unpublished experiments for *Dlac* spore and stalk and vegetative cells and are all listed in Supplemental_Table_S1.xlsx. Washed-out heat maps have maximum read counts equal to or below 10

AATF, also known as Che-1, is a transcription factor, which directly interacts with subunit 11 of human RNA polymerase II, and represses the suppression of growth by the Rb retinoblastoma protein (Fanciulli et al. 2000). A single ortholog was identified in each of the tested Dictyostelid genomes and the gene is most highly expressed during growth.

The ARID (AT-rich interaction domain) is a DNA-binding protein, also known as BRIGHT from the mouse B-cell regulator of IgH transcription (Herrscher et al. 1995). The ARID domain consists of six α-helices and a β-hairpin (Iwahara et al. 2002), and proteins that contain the ARID domain regulate gene expression throughout animal development and participate in chromatin remodelling (Wilsker et al. 2005). *Ddis* has two proteins with the ARID domain, one, RbbB, with an additional JmjC transcription factor domain and the other with a SAP30-Sin3 binding domain. The SAP30-Sin3 complex is associated with histone deacetylases and acts as a corepressor, capable of gene silencing (Grzenda et al. 2009). Both *Ddis* proteins are conserved throughout Dictyostelia and except *Ddis*, the tested species also have a third ARID domain containing protein.

Figure S2. AT-hook DNA binding domain

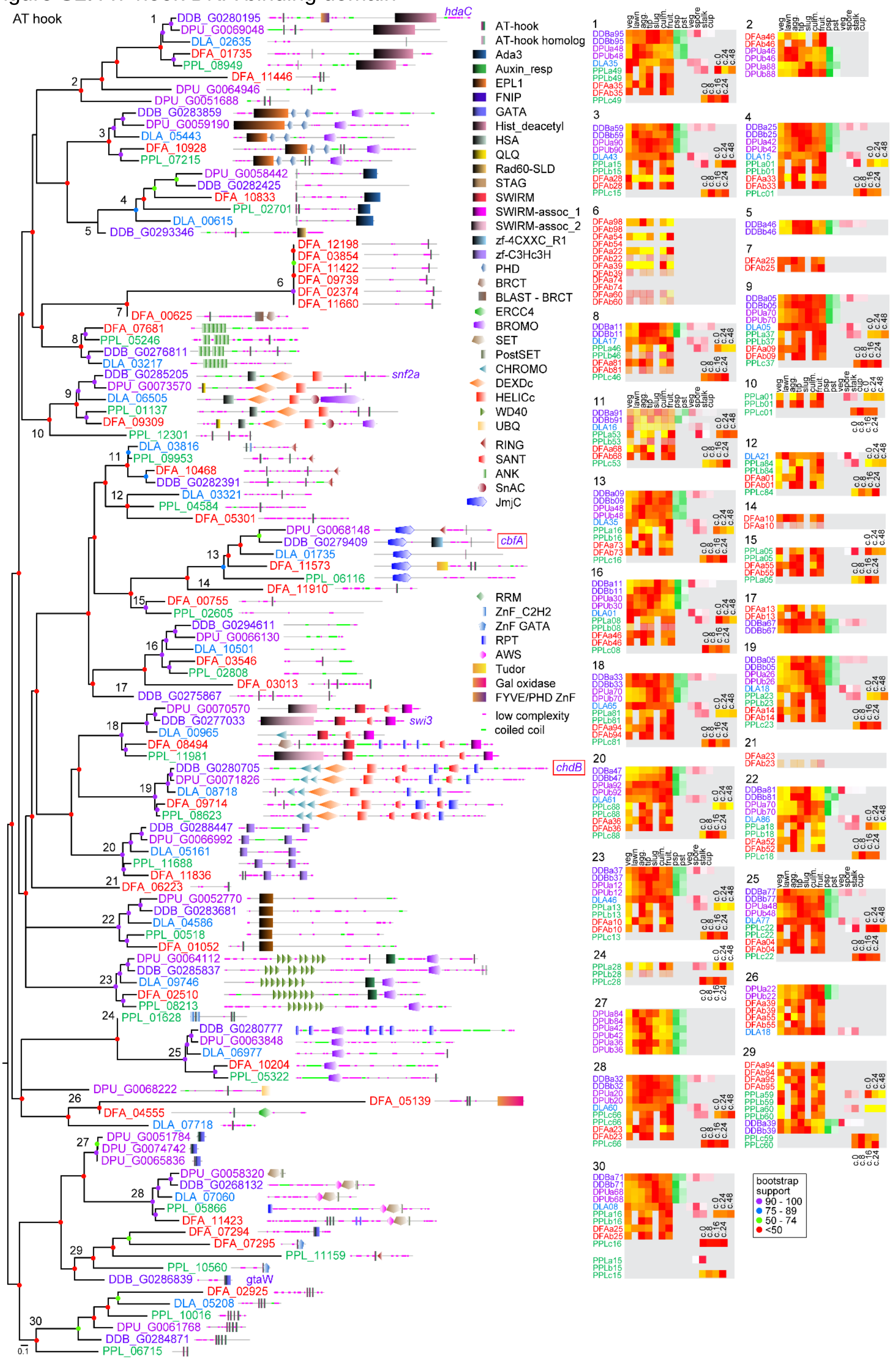

Figure S2. AT-hook binding domain

Sequences with AT-hook domains were retrieved from Dictyostelid genomes with the Interpro identifier (IPR017956). The sequences corresponding to the AT-hook domains and 20 AA of flanking sequence were aligned and a phylogeny was constructed using RAXML. Many tree nodes are unresolved, because due to its small size (11 or 12 AA) the AT-hook domain contains insufficient phylogenetic signal. For orthologous sets that appeared incomplete, missing proteins with retrieved by BlastP search using one of the orthologs as query. The hits often lacked an AT-hook domain recognized by SMART, although most of the underlying sequence was usually present. Minitrees were prepared for the full sets of orthologs, which were grafted onto the main tree to replace the incomplete set. The final tree was annotated with protein domain architectures and transcription profiles as described in the legend to figure S1.

The AT-hook is a small DNA-binding motif that preferentially binds to the minor groove of A/T rich DNA. They are present in mammalian HMGI/Y nuclear proteins that participate in inducible transcription and other interactions with chromatin (Reeves and Beckerbauer 2001), in plant DNA binding proteins (Nieto-Sotelo et al. 1994) and in hBRG1, a component of the SWI/SNF chromatin remodeling complex (Singh et al. 2006). Dictyostelia have a total of about 56 AT-hook proteins, amongst which several chromatin remodelling proteins, such as hdaC, snf2A, swi3 and chdB. In addition, cbfA, a jmjC type TF, contains AT-hook homologous sequence at its C-terminus, which is identical to the GRP domain that was previously shown to mediate cbfA binding to A/T rich regions in the C-module of DRE retrotransposons (Horn et al. 1999). No biological roles are known for the remaining AT-hook proteins.

Figure S3. bZIP

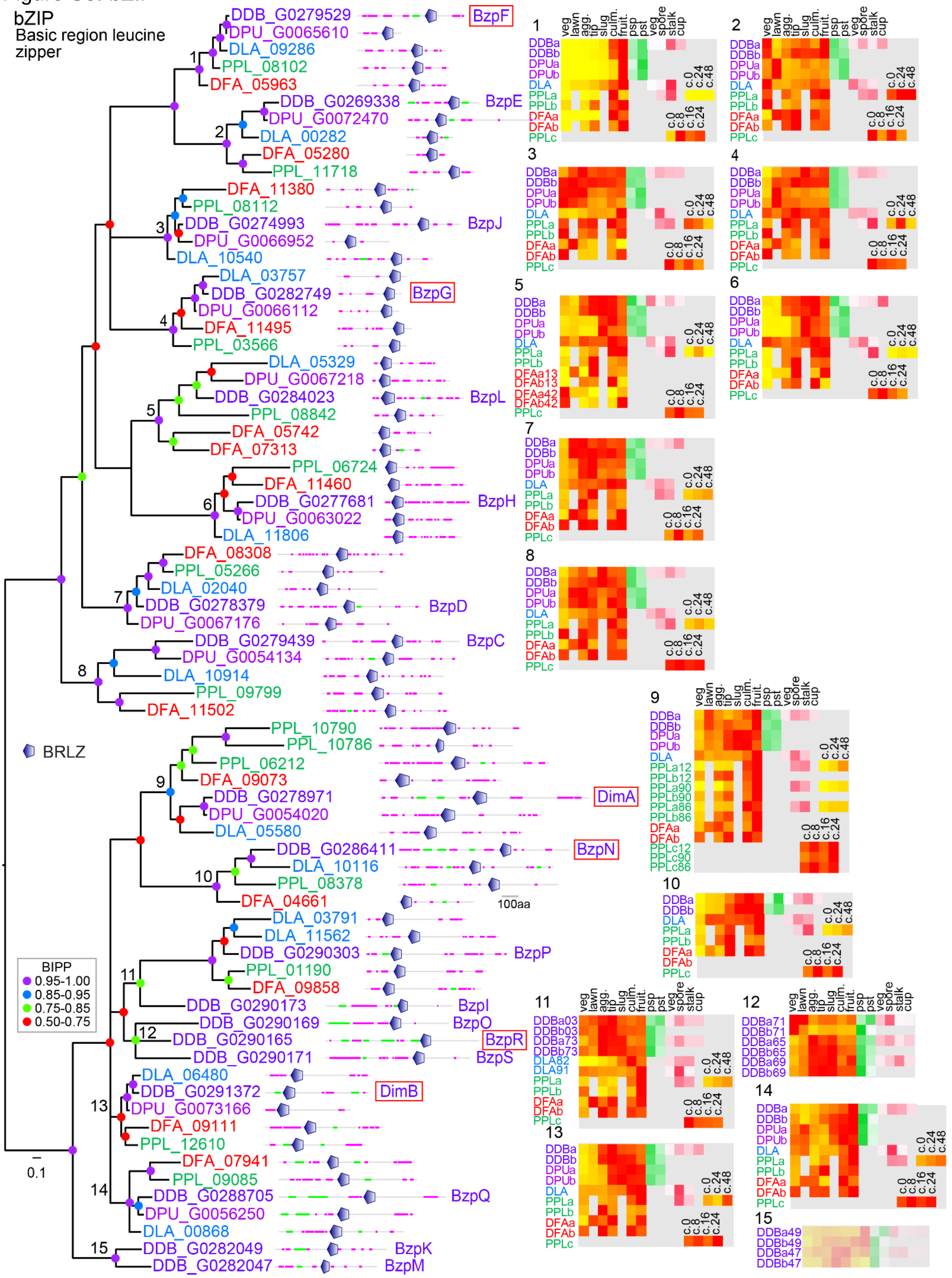

Figure S3. Basic Leucin Zipper Transcription Factors (bZIPs)

Sequences with BRLZ domains were retrieved from Dictyostelid genomes with the Interpro BRLZ domain identifier (IPR004827) and by BlastP and tblastn queries with the sequences of the 19 *Ddis* bZIP factor proteins. The sequences corresponding to the BRLZ domains were aligned and a phylogeny was constructed and annotated with protein domain architectures and transcription profiles as described in the legend to figure S1.

The eukaryotic basic-leucine zipper (bZIP or BRLZ)) transcription factors are able to form both homo- and heterodimers (Hai and Curran 1991). bZIPs contain a basic region mediating sequence-specific DNA-binding followed by a leucine zipper region required for dimerisation. The dimerisation specificity of the leucine zipper allows for combinatorial interactions that can alter DNA binding and thus transcriptional regulation. Of the 19 bZIPs in *Ddis,* six have been functionally analysed. DimA and DimB function in DIF-1 signal transduction (Huang et al. 2006), BzpN regulates cell-density regulated proliferation (Phillips et al. 2011) and BzpF regulates spore maturation and stability (Huang et al. 2011). BzpF is also implicated as a cAMP response element-binding protein (CREB) by transcriptional network analysis, and binds to the canonical cAMP response element *in vitro* (Parikh et al. 2010a). The *bzpG* and *bzpR* genes have also been disrupted, but did not show any observable phenotype (Parikh et al. 2010a).

We find orthologs in *Dfas*, *Ppal, Dlac* and *Dpur* for 13 of the *D.discoideum* bZIPs. For all but three, *bzpC*, *bzpN* and *bzpP*, orthologs were present in all five Dictyostelid genomes. Several bZIPs (*bzpM, bzpK, bzpI, bzpO, bzpT* and *bzpD*) are only present in *Ddis,* while *Dfas* has an additional *bzpI* paralog and *Ppal* two additional *dimA* paralogs*.*

Figure S4A. C2H2 ZnF set 1

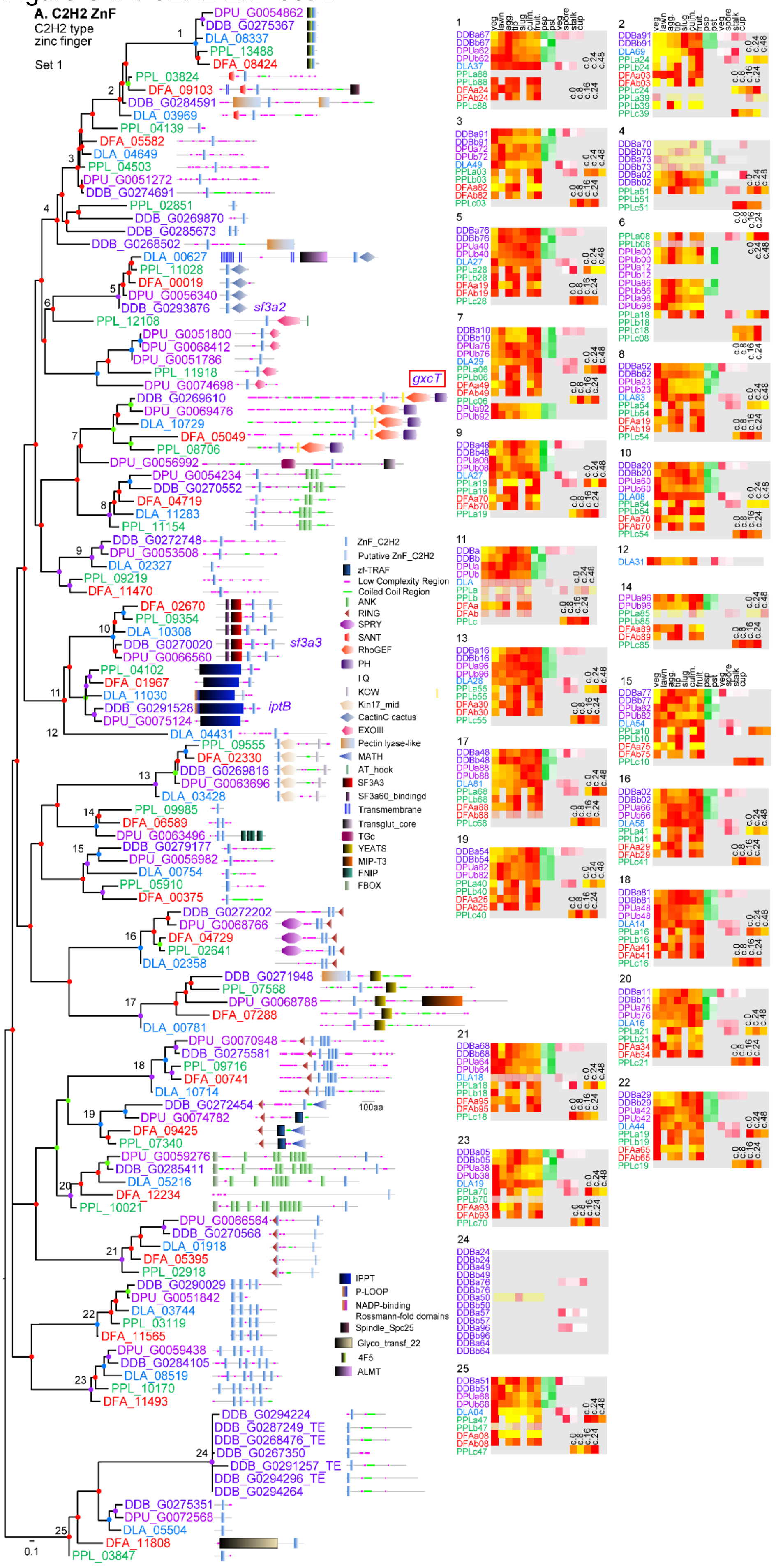

Figure S4B. C2H2 ZnF set 2

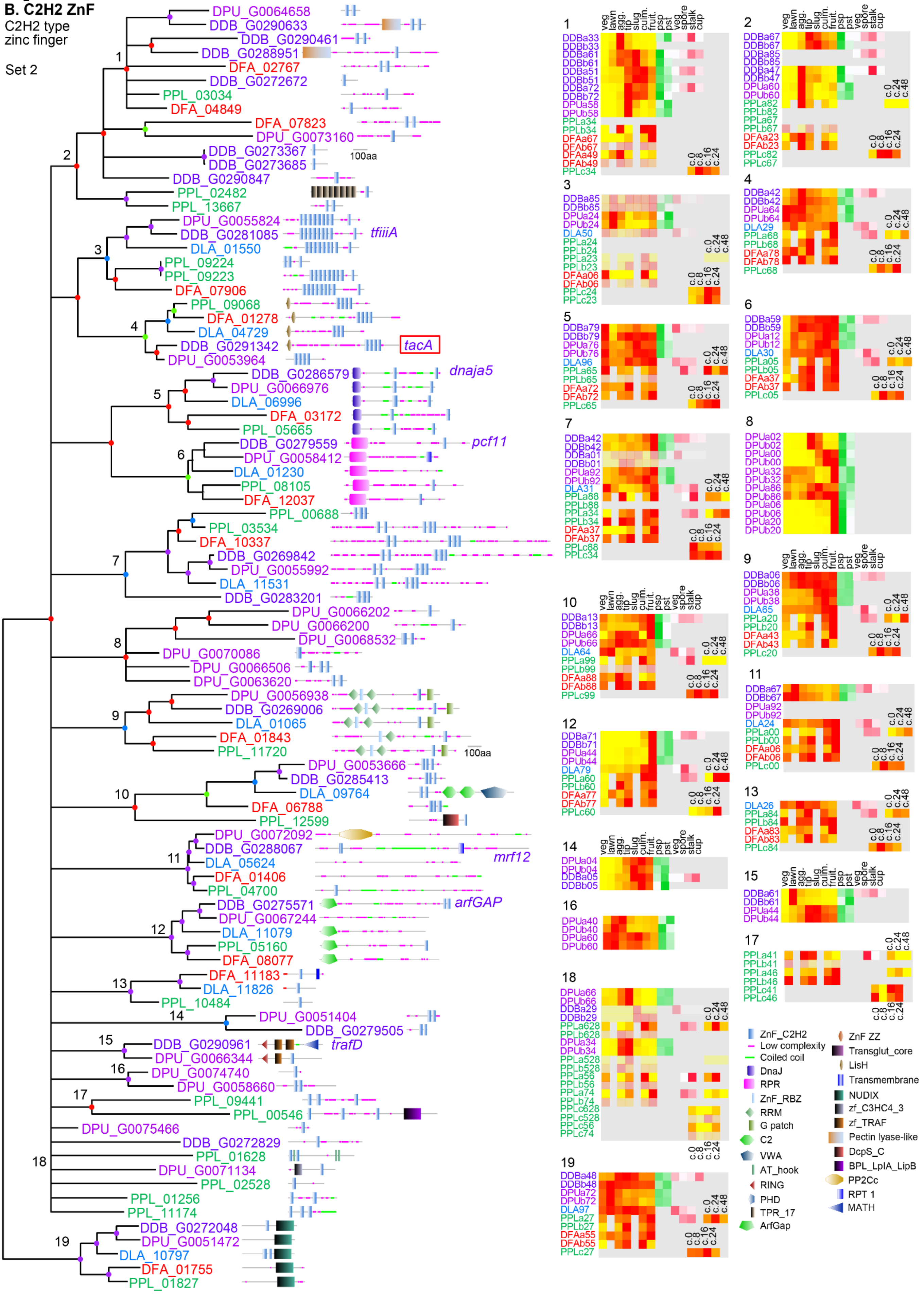

Figure S4C. C2H2 ZnF set 3, C2H5 and CXC.

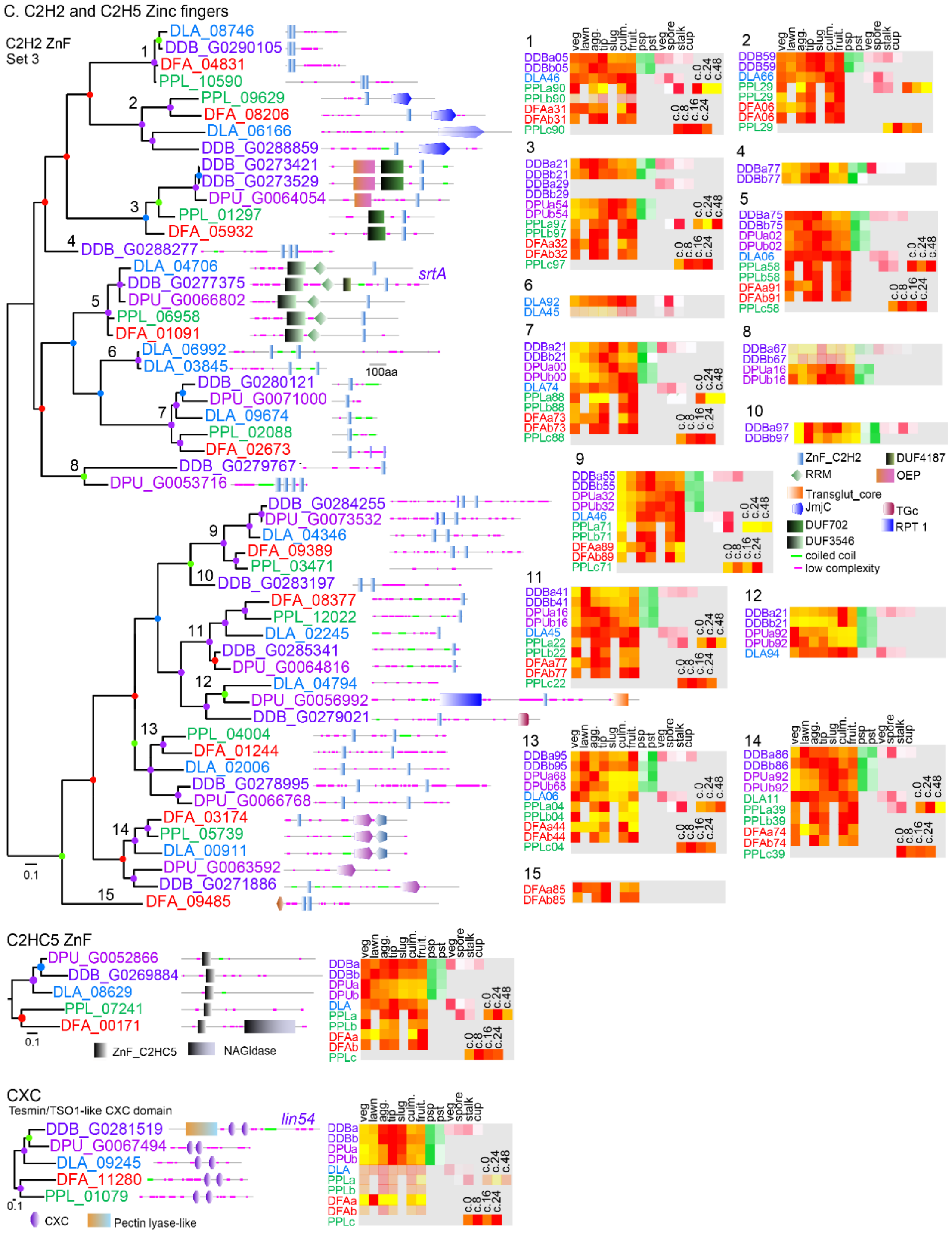

Figure S4. C2H2, C2H5 and CXC zinc finger proteins

Sequences containing the C2H2_ZnF domain were identified from Dictyostelid genomes by the IPR013087 Interpro identifier and by BLAST queries with C2H2 domain sequences. The sequences corresponding to the 23 AA C2H2 domain were aligned and a pilot tree was constructed using RAXML, which was used to subdivide sequences into 3 sets of related proteins. Individual trees were inferred from these sets using RAXML or MrBayes. For orthologous sets that appeared incomplete, missing proteins with retrieved by BlastP search using one of the orthologs as query. Minitrees were prepared for the full sets of orthologs, which were grafted onto the main tree to replace the incomplete sets. The final tree was annotated with protein domain architectures and transcription profiles as described in the legend to figure S1.

Query with the C2HC5_ZnF Interpro identifier IPR009349, the CXC Interpro identifier IPR033467 and Blast searches revealed only a single well conserved gene for each in Dictyostelia.

The C2H2 (Cys_2His_2) zinc finger was first identified in TFIIIA (Miller et al. 1985) and is common to eukaryote transcription factors. However, C2H2 Zn finger proteins can also bind to RNA or to proteins, either alone, or in addition to DNA binding. The C2H2 Zn finger consists of two β-strands and an α-helix, which is

stabilised by the binding of a zinc ion, with two conserved cysteine residues at one end of the β sheet and with two conserved histidine residues at the α-helix C-terminus. It associates with the major groove of DNA and commonly acts in tandem repeats in sequence-specific DNA binding (Iuchi 2001).

Dictyostelia contain 103 different genes with C2H2 domains and most of them are conserved in all 4 taxon groups. Only two genes, *tacA* (set 2) and *gxcT* (set 1) were functionally analysed in *Ddis*. TacA is a transcription factor which translocates to the nucleus in a Ca^{2+} and calcineurin dependent manner. *TacA* silencing results in delayed development and formation of smaller fruiting bodies with only partially ascended spore heads (Thewes et al. 2012). The nucleotide exchange factor, gxcT, is involved in stabilizing PIP3 production in response to chemoattractants and hence controlling stable spatial sensing during chemotaxis. (Wang et al. 2013). This gene is obviously not a transcription factor, highlighting that some of the *Ddis* C2H2 proteins are likely not directly involved in gene regulation.

The C2HC5 zinc finger was previously detected in the thyroid receptor interacting protein 4, which becomes a strong transcriptional activator when fused to the yeast LexA repressor (Lee et al. 1995). *Ddis* has only a single well conserved C2HC5 containing protein, which has not been functionally analysed.

The CXC family has two copies with the zinc binding cysteine-rich motif C-X-C-X4-C-X3-YC-X-C-X6-C-X3-C-X-C-X2-C and is found mammalian tesmin (Sugihara et al. 1999), its *C. elegans* ortholog LIN54 (Harrison et al. 2006) and *A. thaliana* TSO1 (Andersen et al. 2007). Dictyostelia contain a single conserved lin54 ortholog that is transiently expressed in group 4 prespore cells, but poorly in other groups.

Figure S5. CBF/NF-Y/Archaeal Histone

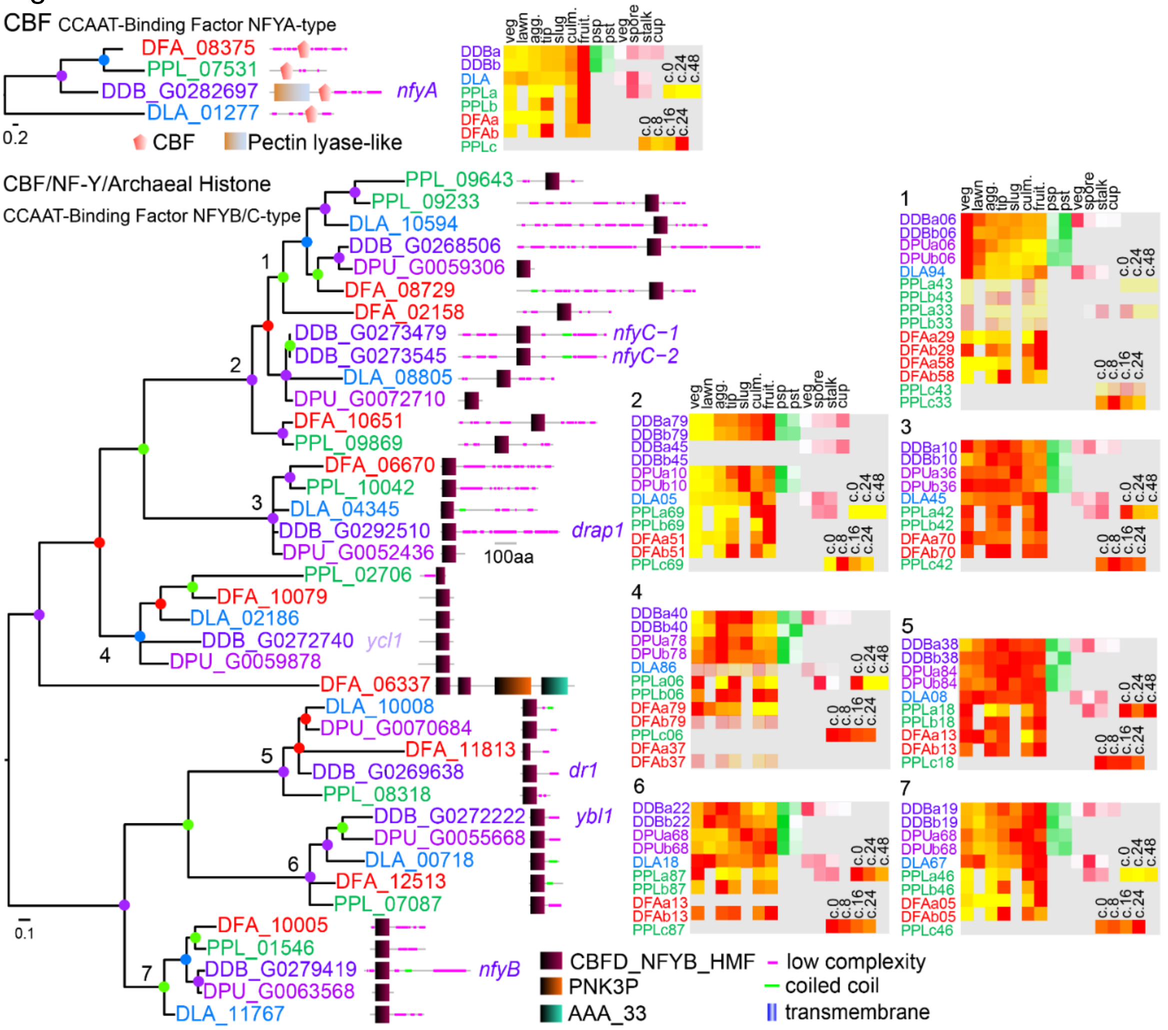

Figure S5. CBF/NF-Y/Archaeal Histone transcription factors

Sequences with NF-YA CCAAT and NF-YB/NF-YC CCAAT binding domains were identified with Interpro identifiers IPR001289 and IPR003958, respectively. Genomes were queried further by BlastP and tblastn with the retrieved sequences. Annotated trees were constructed as described in Figure S1.

The CCAAT-binding factor(CBF)/Nuclear transcription factor Y(NF-Y)/archaeal histone factor comprises of three subunits, NF-YA, NF-YB, and NF-YC, which specifically bind to CCAAT sequences in promoter regions. The NF-YB and NF-YC subunits dimerize through their histone-fold motifs, and associate with NF-YA to form the trimeric complex (Mantovani 1999). YBL1 and YCL1 share sequence similarity to NF-YB and NF-YC, respectively, but the YBL1-YCL1 dimer does not bind to NF-YA or interact with CCAAT sequence (Bolognese et al. 2000). The NF-YB/C-like proteins Dr1 and Drap1 associate to repress transcription by preventing formation of the preinitiation complex (Mermelstein et al. 1996).

The *Ddis* genome contains one *nfyA* homolog, which is conserved in all taxon groups and 8 nfyB/C homologs, amongst which *nfyB*, *nfyC*, *ybl1*, *drap1* and *dr1*, all well conserved throughout Dictyostelia. There is no annotated *ycl1*, but sequence similarity suggests that DDB_G0272740 is an *ycl1* ortholog. Additionally DDB_G0268506 is an *nfyB/C* type gene that is well conserved in Dictyostelia. None of the *Dictyostelium* NFY type proteins have been functionally analysed.

Figure S6. Crtf and cudA-like transcription factors

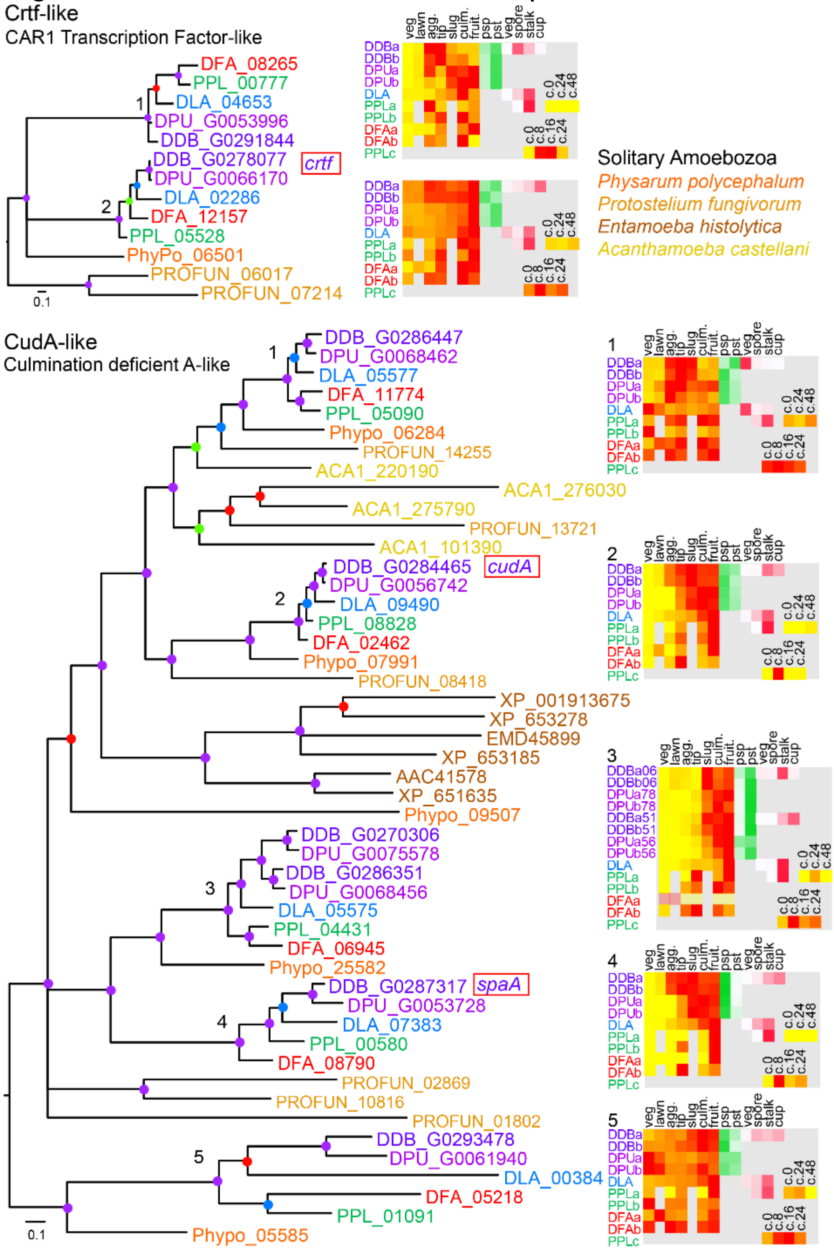

Figure S6. CudA-like transcription factors

Crtf and cudA are transcription factors that were first identified in Dictyostelia. They have previously annotated functional domains or Interpro identifiers. We retrieved homolog and orthologs of these TFs by BLASTp searches with *Ddis* crtf and cudA sequences. Because it is unknown whether these TFs are also present outside of Dictyostelia, we also performed BLASTp queries of Genbank and Amoebozoan genomes. Both crtf and cudA-like proteins were found in other Amoebozoa, but not in other eukaryotes. Phylogenetic trees were constructed using MrBayes from unambiguously aligned regions of protein sequence. Gene IDs and locus tags are colour coded to reflect host species and gene expression profiles were added as described for Figure S1.

Crtf is a transcription factor with a zinc finger-like motif that binds to the cAMP receptor 1 (cAR1) promoter and is essential for cAR1 expression and spore maturation (Mu et al. 2001). There is one crtf homolog in *Ddis* and both proteins are conserved throughout Dictyostelia. Crtf-like proteins were also detected in solitary Amoebozoa.

CudA is a transcription factor, which is expressed in both the prespore region and the tip of slugs. (Fukuzawa et al. 1997; Yamada et al. 2008). Tip-specific expression is required for initiation of fruiting body formation, while prespore-specific expression is essential for expression of some prespore genes. SpaA is essential for spore maturation and expression of PKA regulated prespore and spore genes (Yamada et al. 2018). *Dictyostelids* have 5 conserved cudA-like genes, of which one is duplicated in group 4. Several CudA-genes also have orthologs in solitary Amoebozoa.

Figure S7. E2F/DP, EnY2, FAR1 and Gal4-like transcription factors

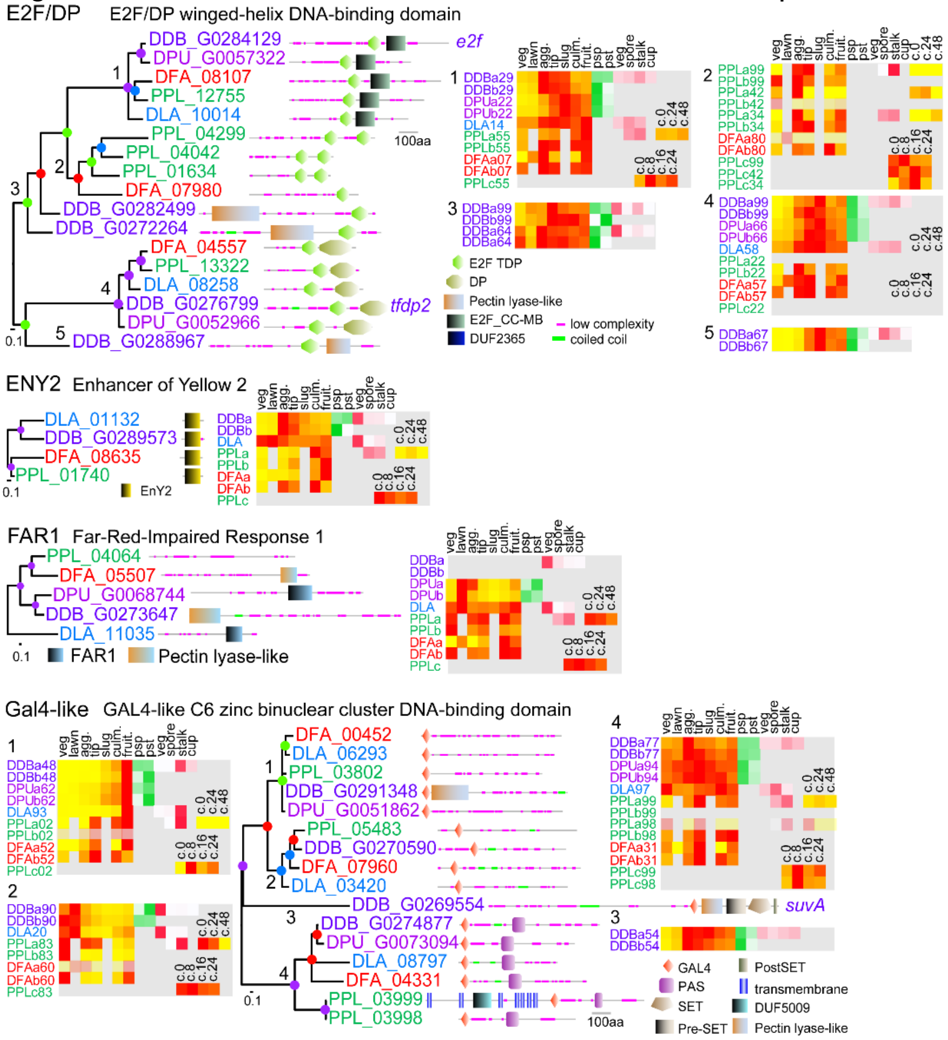

Figure S7. E2F/DP, ENY2, FAR1 and Gal4-like transcription factors

The sequences containing an E2F/DP, ENY2, FAR1 or Gal4-like domain were retrieved from *Dictyostelia* by the Interpro identifiers IPR003316, IPR018783, IPR004330 or IPR001138, respectively, and by performing BlastP searches. Sequences were aligned for Bayesian phylogenetic inference and trees were annotated as described for Figure S1.

E2F (adenovirus early gene 2 promoter-binding factor) and DP (dimerization partner) both display an N-terminal winged-helix fold for DNA-binding and a C-terminal dimerization domain. E2F proteins can form homodimers, but heterodimerization with DP increases DNA-binding efficiency (Zheng et al. 1999). E2F promotes the G1/S transition by activating transcription of cell cycle control genes and is itself negatively regulated by the retinoblastoma protein (Wu et al. 1995). The *Ddis* genome contains five proteins with the E2F TDP domain, including *e2f* and *tfdp2*. Both genes are conserved throughout Dictyostelia, with a second tfd2-related gene in *Ddis*. Their expression increases after starvation, particularly in prespore cells. In addition there is a small cluster of e2f-like genes unique to taxon groups 1 and 2 and a set of 2 genes unique to *Ddis*.

The enhancer of yellow 2 (EnY2) acts as a transcription factor in *Drosophila* (Georgieva et al. 2001), but is also known as SUS1 in yeast, where it is involved in mRNA export coupled transcription by interacting with the SAGA and TREX2 complexes (Pascual-García et al. 2008). Dictyostelia have a single *eny2* gene that is conserved in all taxon groups. FAR1 is a plant transcription factor, related to mutator-like transposases, which is required for responses to far red light (Lin et al. 2008). Although the FAR1 domain was detected only in *Dlac* below threshold levels, its sequence is also well conserved in the other Dictyostelia.

The yeast Gal4 transcription factor contains a Zn(II)2C6 type binuclear cluster with six cysteines that interact with two zinc atoms (Pan and Coleman 1990). The zinc cluster can bind to CGG triplets or direct, inverted or everted CGG repeats as monomers, homodimers and heterodimers and are considered to be unique to fungi (MacPherson et al. 2006). Dictyostelia contain 3 deeply conserved genes with a GAL4 domain, and one gene, *suvA*, unique to *Ddis*. The *Drosophila* homolog of this gene, which does not have a GAL4 domain, is involved in heterochromatin mediated gene silencing (Schotta et al. 2003).

Figure S8A. GATA ZnF set 1

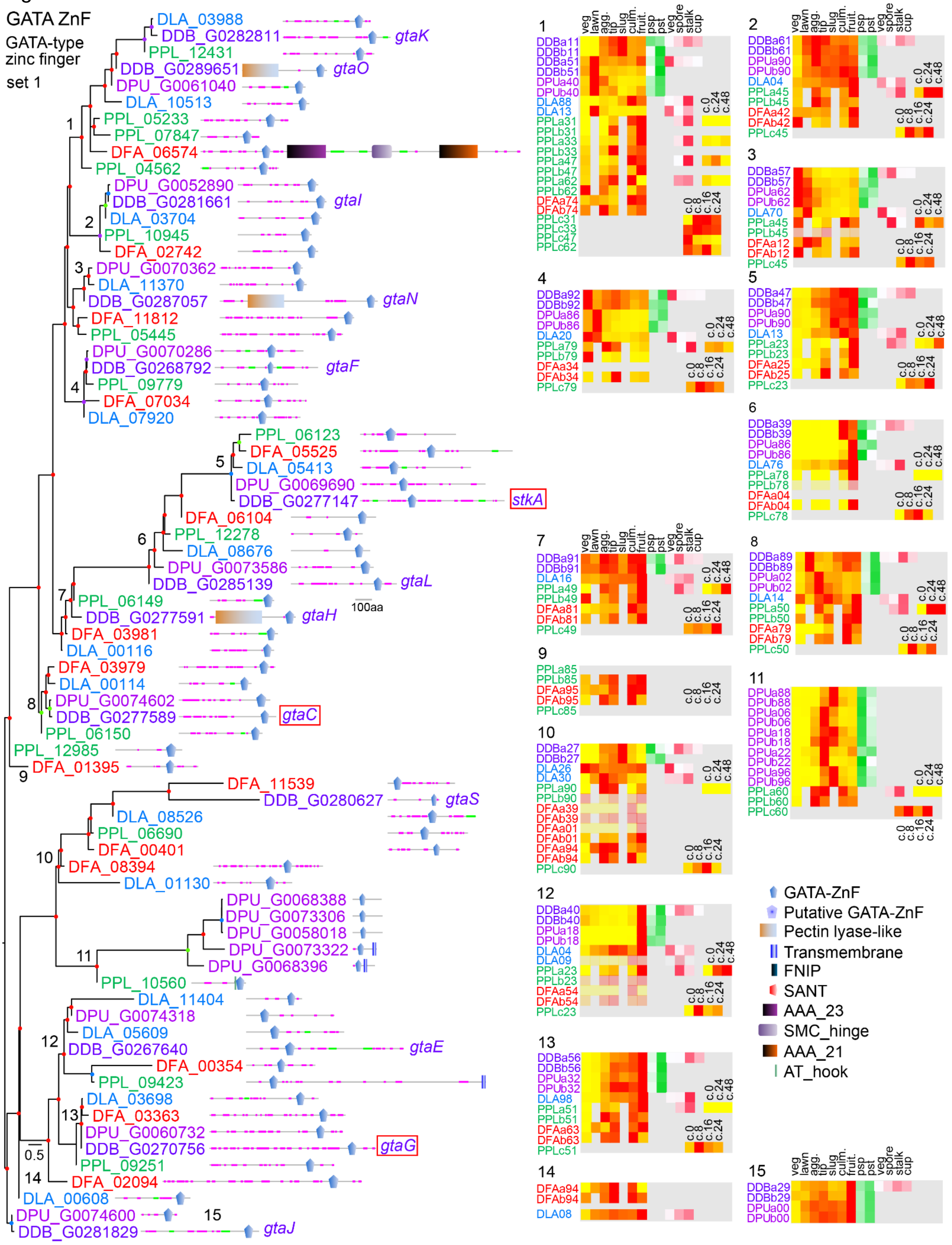

Figure S8B. GATA ZnF set 2

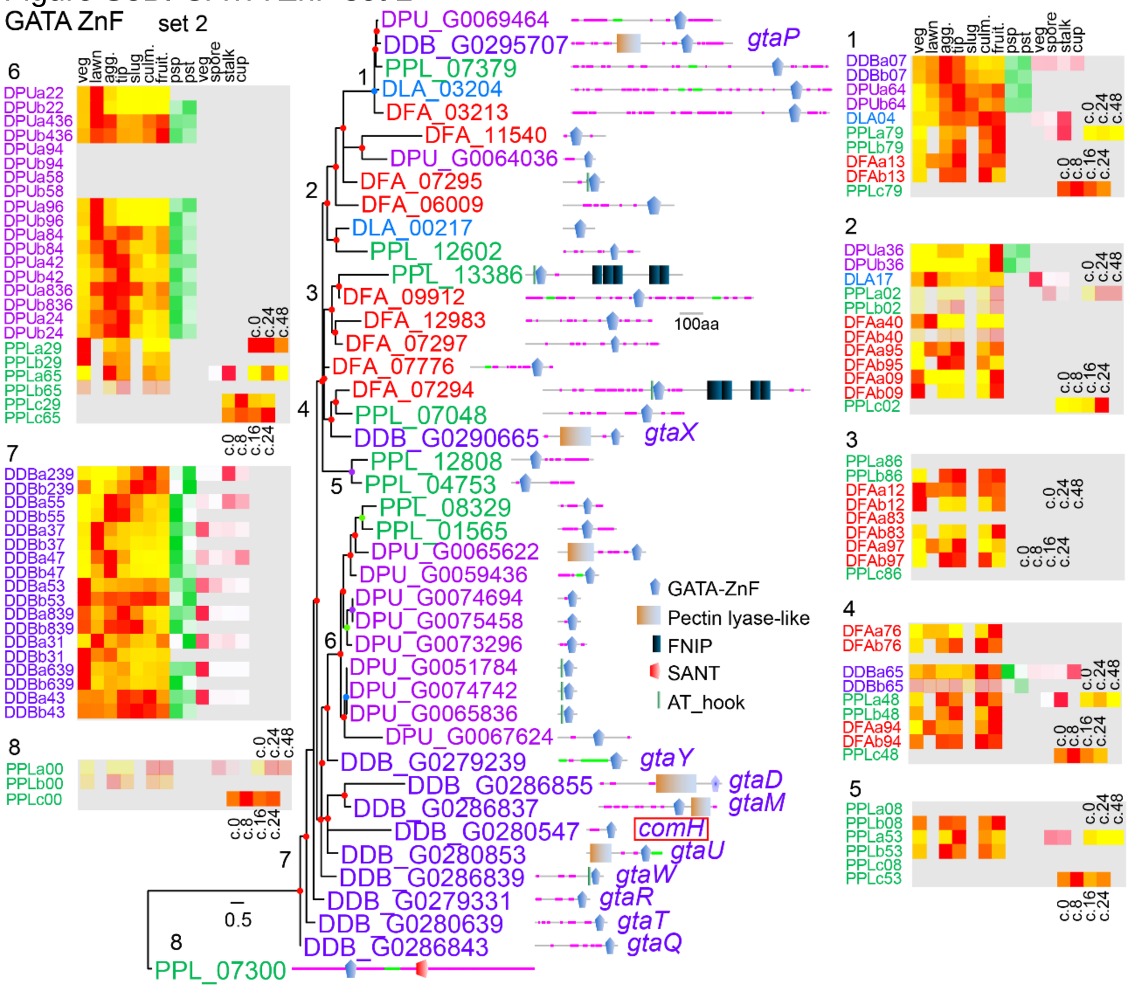

Figure S8. GATA-type zinc finger transcription factors

GATA-ZnF domain containing proteins were identified in Dictyostelid genomes using the InterPro identifier, IPR000679, and by BlastP search. After sequence alignment, a phylogenetic tree was inferred using RaxML. The two main branches of the tree are shown separately as set 1 and set 2.

The GATA-type zinc finger domain is found in many transcription factors and its name is derived from the target DNA sequence (T/A)GATA(A/G) (Yamamoto et al. 1990). The zinc ion is coordinated by four cysteines in a core that consists of two antiparallel beta sheets, an alpha helix and a long loop that connects with the carboxyl-terminal tail (Omichinski et al. 1993). The *Ddis* genome has 24 genes with a GATA ZnF domain, with about 13 genes conserved across Dictyostelia and 9 resulting from *Ddis* specific amplification from a single gene. *Dpur* and to a lesser extent *Ppal* and *Dfas* also show species- or group-specific gene amplifications.

One of the expanded *Ddis* genes is *comH*, which is required for sporulation and development beyond the tight aggregate stage in a non-cell autonomous manner (Kibler et al. 2003). Three of the conserved genes were functionally analysed. Mutants defective in *gtaC* show defective aggregation and lack cAMP pulse induced gene expression. In wild-type, the cAMP pulses cause out-of-phase transport of GtaC to and from the nucleus (Cai et al. 2014; Santhanam et al. 2015). *gtaC-* cells also form fragile slugs and fruiting bodies that fail to remain erect. The latter phenotype is also found in mutants defective in DIF-1 signalling. DIF-1 was found to both induce gtaC expression and its translocation to the nucleus (Keller and Thompson 2008). *GtaG* is expressed in prestalk and stalk cells and *gtaG* null mutants cannot from fruiting bodies. This defect is rescued by the stalk inducing factor c-di-GMP, suggesting that GtaG is (indirectly) required for c-di-GMP synthesis (Katoh-Kurasawa et al. 2016). StkA is required for terminal spore differentiation and in *stkA*-mutants the prespore cells differentiate into vacuolated stalk (Chang et al. 1996).

Figure S9. G-box binding factor

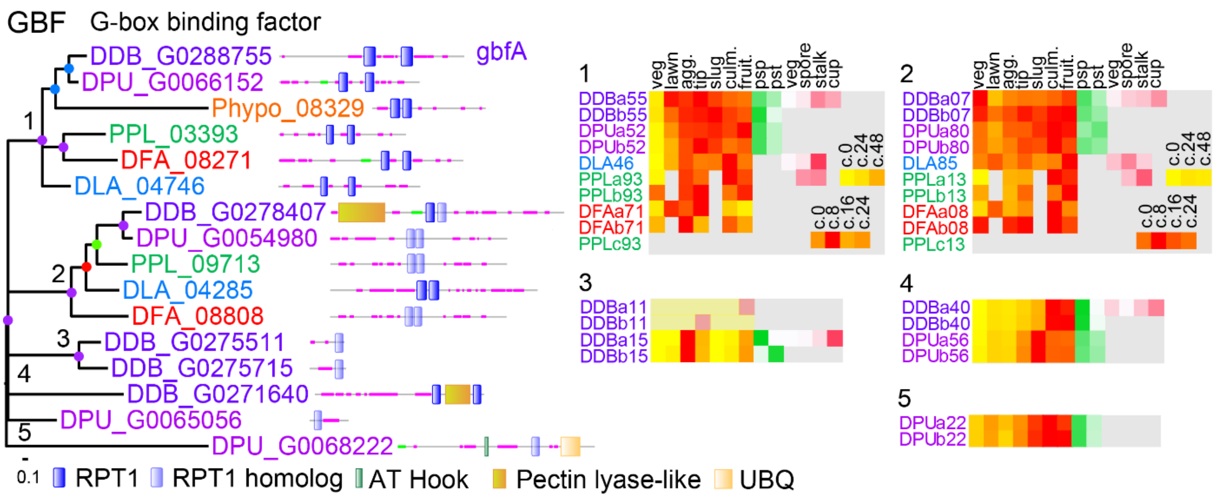

Figure S9. G-box binding factor

The G-box binding factor *gbfA* was identified in *Ddis* as a protein binding to GC rich regions in the cAMP-inducible gene cprA (Hjorth et al. 1990; Schnitzler et al. 1994) and was shown to be essential for cAMP induction of cprA and other cAMP-inducible genes. GbfA contains two putative zinc fingers and its expression is itself upregulated by cAMP (Hjorth et al. 1989; Brown and Firtel 2001). We searched for gbfA homologs in Amoebozoa using BlastP with gbfA as bait and identified orthologs of gbfA and another gbf-like protein in all Dictyostelia as well as respectively three and two more homologs in *Ddis* and *Dpur.* The putative zinc fingers with their two CxxC motifs were recognized in most proteins by SMART as RPT (repeat) 1 domains, but were also present when no RPT1 domain was recognized, as shown in the alignment below. A gbfA ortholog was also detected in the Amoebozoan *Physarum polycephalum.*

CxxC (RPT1) domain 1

```
DDB_G0288755  GQTIPKCTRCNEAASWKHDK--RRWWCKECKKAFTPGITKMQQVPQQAQLQPLQNHNQIIPQLWDSQQNN 402
DPU_G0066152  NGTIPKCTRCNESASWKHDK--RRWWCKECKKAFTPGITKMQQVPQQVQLQPLQNHAQMIPQLWESNSSQ 296
Phypo_08329   SRKVPSCTRCAARAAWKHDKKGKRWFCKICKLPFTPGVSPAHKLK------------------------- 112
PPL_03393     PNTIPKCTRCSESASWRHDK--RRWWCKECKKAFTPGISKLQNA----------SPTP-------QTQMP 227
DFA_08271     PLVIPKCTRCNESASWRHDK--RRWWCKECKKAFTPGITKSPSA----------STPSPQPLLQSPLPMG 446
DLA_04746     PVSIPKCTRCNENASWRHDK--RRWWCKECKKAFTPGITKNQNTQQVQTIQNPQLPTNPMPQGLMYQGQM 224
DDB_G0278407  VNENPACPKCSIPATRRHDK--RRWWCKPCSKSFTPFRKEKRLSN------------------------- 407
DPU_G0054980  LNENPSCPKCNVPATRRHDK--RRWWCKPCTKSFTPFRKEKRLSN------------------------- 323
PPL_09713     LGENPACPNCNIPATRRHDK--RRWWCKNCTKSFTPFRKGKKLDA------------------------- 319
DLA_04285     TEENPCCPKCTTPATRRHDK--RRWWCKICTKSFTPYRKEKKAML------------------------- 377
DFA_08808     QMDKPTCPNCHVIATRRHDK--RRWWCKNCVKSFTPFRKGKKYND------------------------- 325
DDB_G0275511  --------------------------------------------------MI--------------NPNF 6
DDB_G0275715  ---------------------------------------------------------------------- 1
DDB_G0271640  VNELPNCAYCNRVASWKHDK--KRYYCRNCNKAFIPIENRIYKR---ATDS---------------EFNI 496
DPU_G0065056  ---------------------------------------------------------------------- 1
DPU_G0068222  PNSATTSPLLSSIPKKRGRP--PKFQAQTAQ-QINNIVANISGKPTSYGDGF--------------SLNE 430
```

CxxC (RPT1) domain 2

```
DDB_G0288755  --------NSLITQNTLNSLSTSVSCPPCPLCRGISSWKHDKKRYFCKECKKPFTPVGAGLSPSSSPSSP 524
DPU_G0066152  ------------NNTQITSPSISGQCPPCPQCRGVSSWKHDKKRYFCKECKKPFTPTGQGLSPSPSSPK- 376
Phypo_08329   ---------------------EVVTIPPCPKCFSNEGWKQEKGRYFCTKCRRSFRPRIARGATRSSARKA 161
PPL_03393     -----------------NPNQVTPVSCNCPSCGGTAGWKHDKKRWFCKDCKKAFTLQPDGSMPTSPTSPK 304
DFA_08271     ---------------------MTASQAICPNCNNPGGWKHDKKRWHCKTCKKPFTPSMHDSISGMPSSPT 519
DLA_04746     --------LSNVMPTNNTNNNVAQVPPTCPNCRGLSGWKHDKKRWYCKECRKPFTPNLSSSNSPSKTKKS 310
DDB_G0278407  ------------------DEILSNVTPQCGRCFNQATRKHDKRRWWCKSCRKPFTPTHFNLSENP-NMIY 458
DPU_G0054980  ------------------DELISNVAPQCGRCLNQATRKHDKRRWWCKFCKKPFTPTHFNLTEPQ-NMIY 374
PPL_09713     -----------------NGEFIGNTAPQCGRCFQQATRKHDKRRWWCKVCKKPFTPSQFNLGDNS-HIIF 371
DLA_04285     ------------------GHEMSNSAPHCGRCYQQATRKHDKRRWWCKECKKPFTPTQFNLSSSDGQQIY 429
DFA_08808     -----------------PNSSNPSLAPQCGRCSTQATRKHDKRRWWCKSCKKPFTPNQFSENSQVIYVVG 378
DDB_G0275511  KDQKKIIEPLKNNNNNKIQIEHLQSNVRCKKCNELPSFKHDNKRWYCKFCEKPFTPISFKNLTTENFQK- 133
DDB_G0275715  KFKQKKISLKKEKSQKKIINDQLKIDIKCKKCNCPPSFRHDKKRWWCGKCEKPFTPISFNILTRDNFEKN 138
DDB_G0271640  YSQNTIITNQNNQNCLNNQSDSLQQIPNCTRCGLAPAWKHDKFRWWCKTCSRAYTPNVVRTGYTLNKV-- 647
DPU_G0065056  ---------MDQTEQQLPPELIKKLQNKCTRCNGLPSHKHDQKRWFCKPCNKPFTPGKHFNLNTSNLSLT 61
DPU_G0068222  TTTTTTANISNNNTPNKKSKGNQSDNPKCTRCNEQSAWKHDKKRWWCKNCKKSFTPGVNPKPYNSVANIG 563
```

Figure S10. GCFC, HLH, HMG and HSF

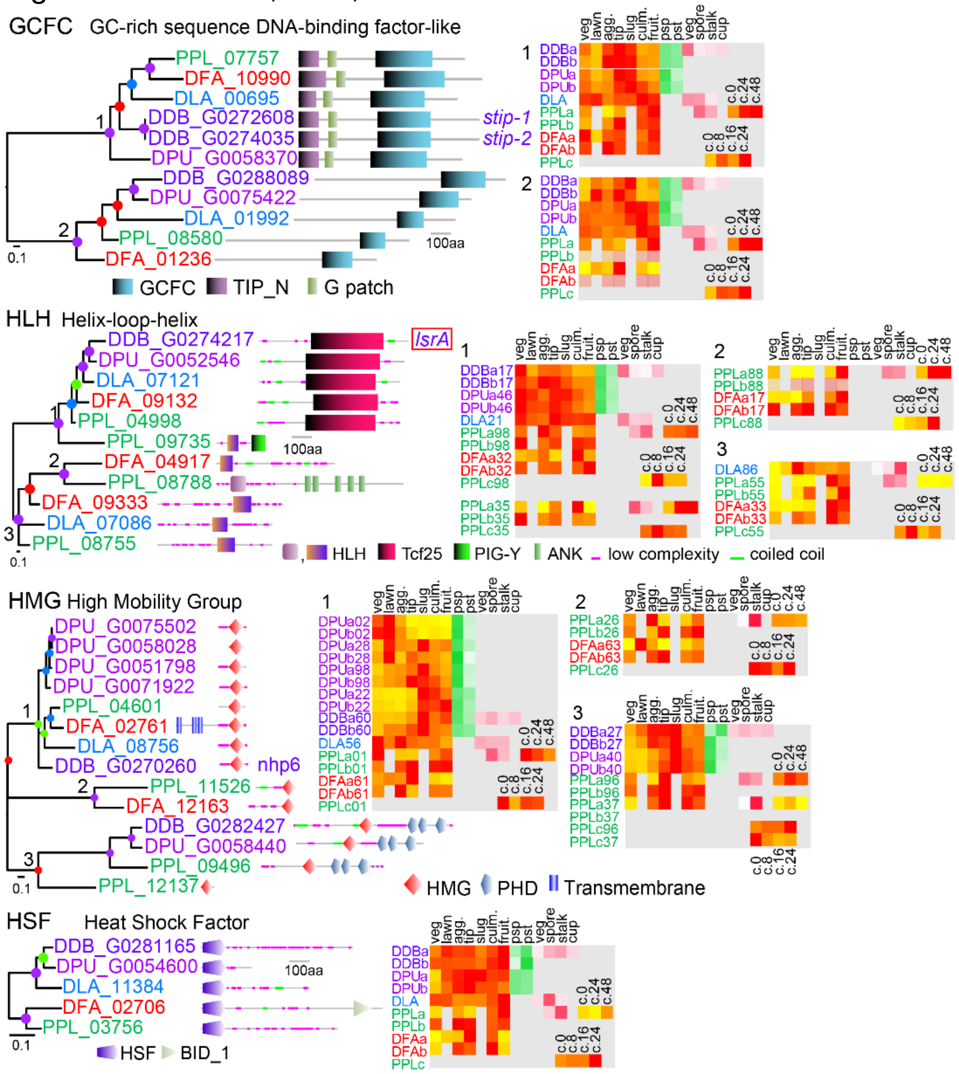

Figure S10. GCFC, HLH, HMG and HSF transcription factors

Sequences with GCFC domains were retrieved with Interpro identifiers IPR012890 and IPR022783. HLH, HMG and HSF proteins were retrieved with identifiers IPR011598, IPR009071and IPR000232, respectively. Dictyostelid genomes were further probed by BlastP using GCFC, HLH, HMG and HSF sequences. Phylogenetic trees were inferred and annotated as for figure S1.

The GC-rich sequence DNA-binding factor (GCFC) domain was originally identified in a transcriptional repressor (Kageyama and Pastan 1989), but is also present in a protein that interacts with the Pax7 transcription factor (Diao et al. 2012) and in septin and tuftelin interacting (stip) proteins that function in RNA splicing (Wen et al. 2005). Dictyostelia contain two conserved GCFC domain proteins; one is homologous to stip (Yu et al. 2011) and the other is of unknown function.

The basic helix-loop-helix (HLH) domain is the DNA binding region of a large family of transcriptional regulators (Jones 2004). *Ddis* has only a single gene, *lsrA,* with Tcf25 repressor region that contains a HLH domain (Cai et al. 2006). LsrA defective mutants were identified as “losers”, because they become overrepresented in the prestalk population when mixed with wild-type cells (Parkinson et al. 2011). *LsrA* is conserved across Dictyostelia, while non-group 4 species have from 1 to 3 other HLH transcription factors.

High mobility group (HMG) domains are present in many transcription factors and other DNA binding proteins involved in replication and repair, and also mediate protein-protein interactions (Stros et al. 2007). Their role in Dictyostelids is as yet unknown.

Heat shock factor (HSF) activates transcription of heat shock genes in response to increased temperature, which induces HSF trimerization and binding to heat shock element (HSE) sequences in promoter regions (Clos et al. 1990). Dictyostelia contain a single well-conserved heat shock factor, which is expressed throughout development.

Figure S11. Homeo domain

HOX Homeodomain transcription factors

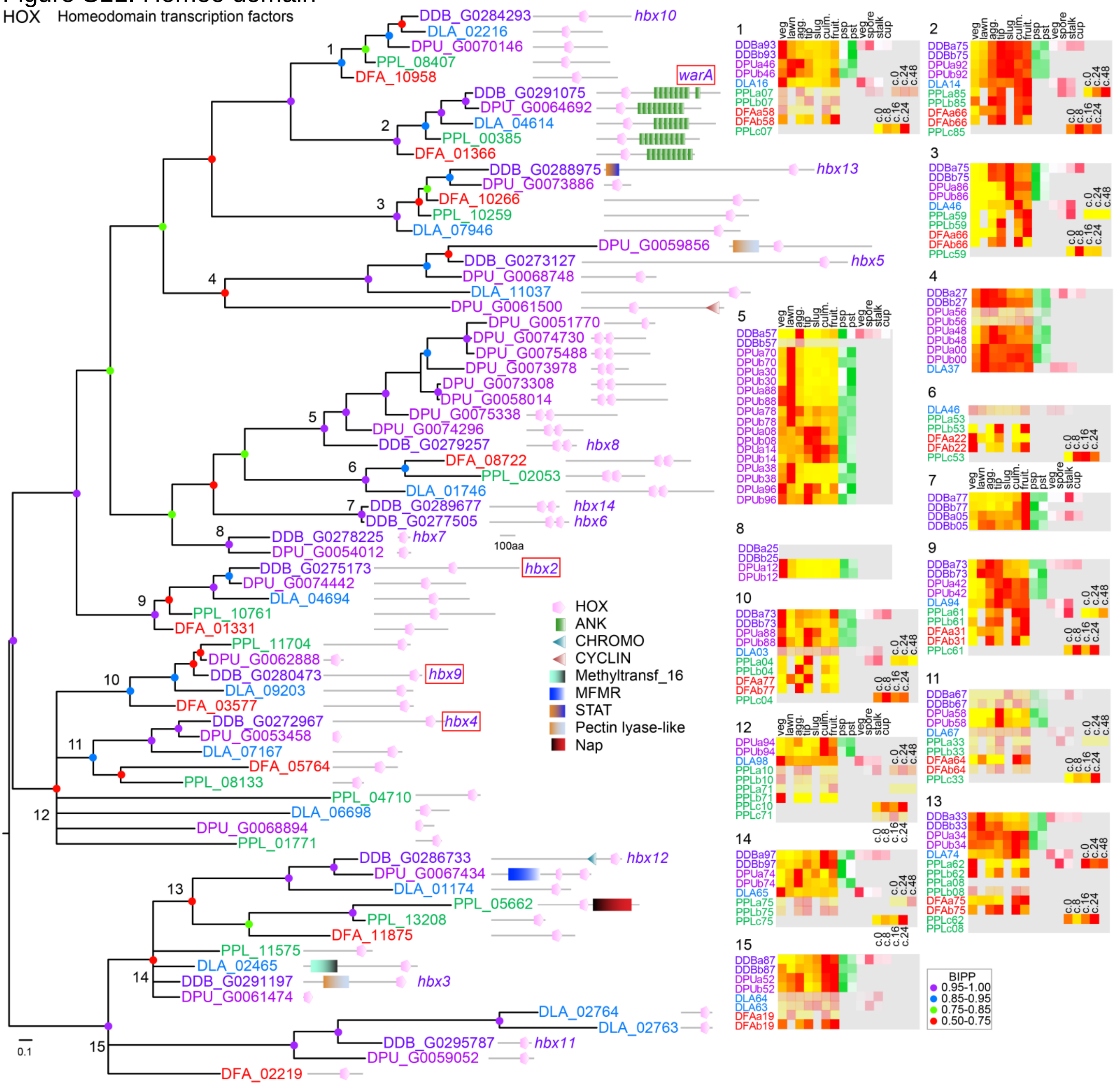

Figure S11. Homeo domain transcription factors

Sequences with homeodomain were retrieved from Dictyostelid genomes with the Interpro identifier IPR001356 and by Blastp search with identified proteins. Phylogenetic trees were inferred and annotated as described in figure S1.

The homeo domain or homeo box (HOX) transcription factors contain helix-turn-helix structure for binding to DNA. They owe their name to the fact that they cause homeotic mutations when mutated in animals, i.e. replace one body part with another, but also have many other functions across phyla (Bürglin and Affolter 2016). The *Ddis* genome contains 14 HOX genes of which 10 are conserved across Dictyostelia. Four genes were functionally analysed. WarA, which also harbours ankyrin repeats, regulates cell type proportioning, most likely by repressing pstO cells (Han and Firtel 1998). Additional knockout of *hbx2* in *warA* null cells slightly enhances the phenotype, and hbx2 may therefore potentiate the function of warA. Overexpression of *hbx4* repressed expression of the Ca^{2+}-dependent adhesion protein cadA (Kim et al. 2011). An *hbx9* knockout shows slow cell proliferation and delayed initiation of development as well as reduced *cadA* expression and overproduction of pstA cells (Mishra et al. 2017).

Figure S12. Jumonji C

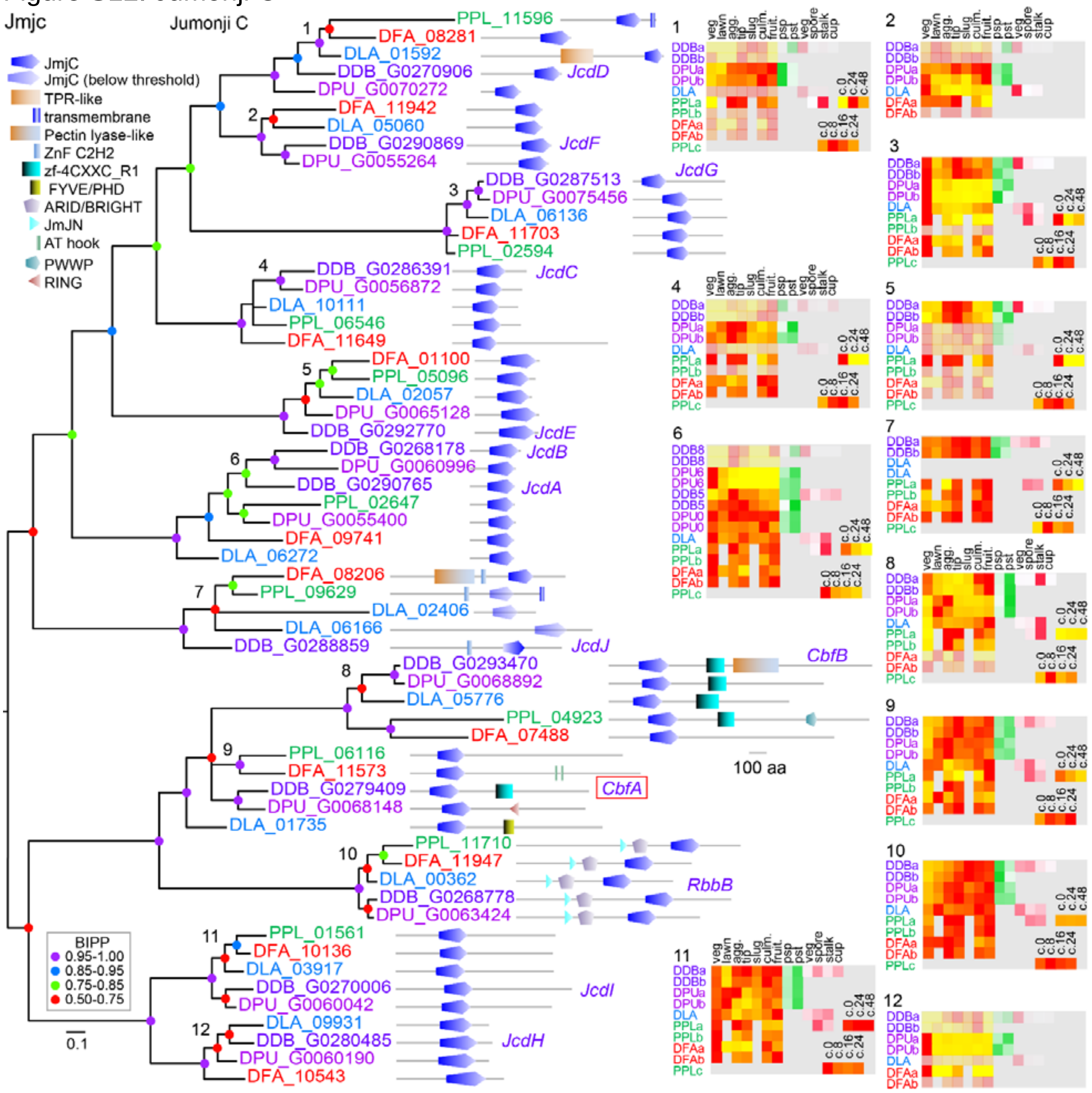

Figure S12. Jumonji C DNA binding proteins

Sequences with jmjC domains were retrieved from Dictyostelid genomes with the Interpro jmjC domain identifier (IPR003347) and by BlastP and tblastn queries with the sequences of the 13 *Ddis* jmjC proteins. Phylogenetic trees were inferred and annotated as described for figure S1.

Jumonji C (JmjC) domains (IPR003347) are found in transcription factors covering a species range from bacteria to humans (Clissold and Ponting 2001). The JmjC domain contains a cupin fold with putative Zn^{2+} binding region that was shown to participate in histone demethylation by hydroxylation (Trewick et al. 2005), which classifies this group of transcription factors as chromatin remodeling proteins. The JmjC domain is often found in combination with an N-terminally located JmjN domain and acts in this configuration as a transcriptional repressor (Takeuchi et al. 2006).

The *Ddis* genome contains 13 genes encoding proteins with JmjC domains and 9 of those were conserved across the four taxon groups. Only one of the *Ddis* JmjC proteins has been examined in more detail. CbfA is a transcription factor activator that binds to the regulatory C-module of the retrotransposon TRE5-A and to an A/T-enriched motif in the promoter of adenylate cyclase A. CbfA is essential for basal but not cAMP-pulse induced expression of ACA (Winckler et al. 2004; Siol et al. 2006) and regulates expression of about 160 genes in the growth phase of development. The *Ppal CbfA* ortholog was shown to functionally complement a *Ddis cbfA* null mutant, indicating that the functions of *CbfA* are evolutionary conserved within Dictyostelids (Schmith et al. 2013).

Figure S13. Lambda, MADS and MIZ/NSE

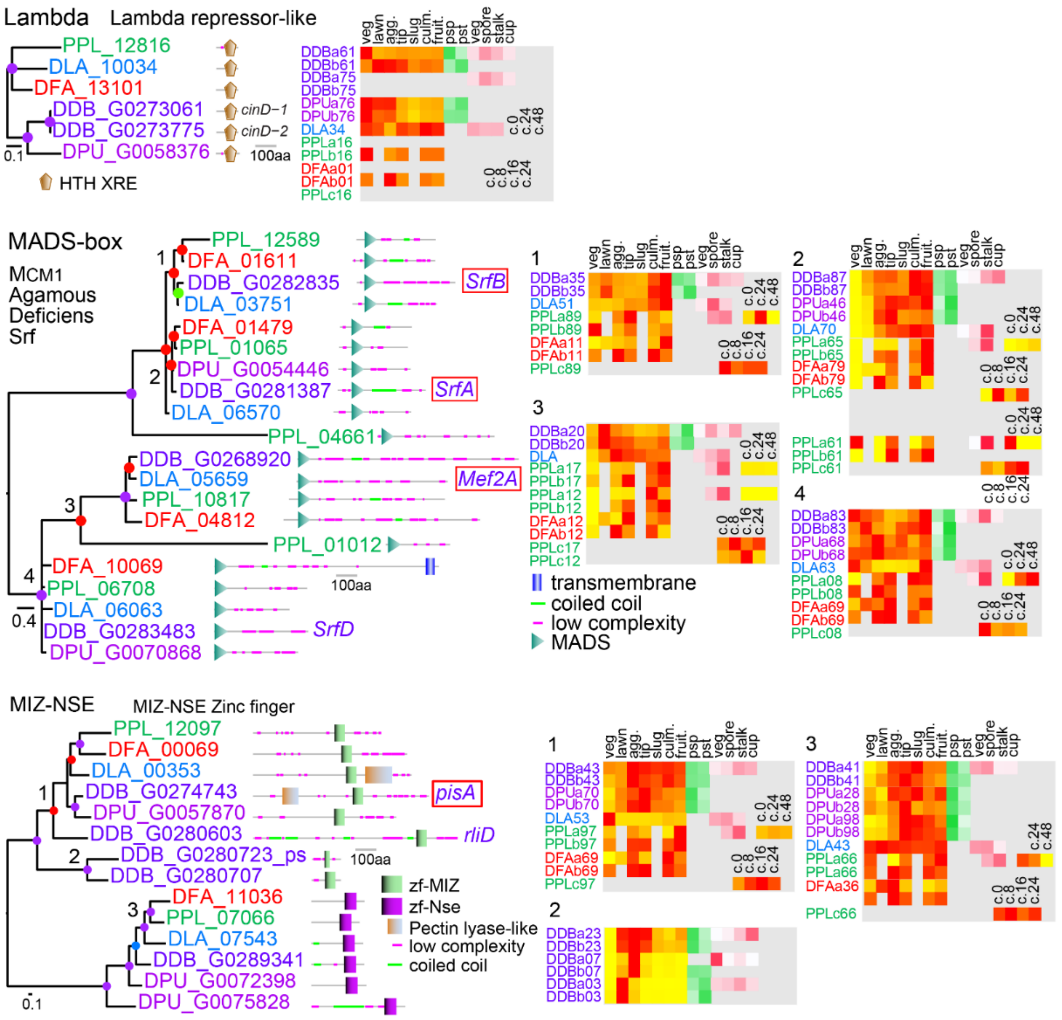

Figure S13. Lambda, MADS and MIZ_NSE DNA binding proteins

Proteins with the lambda repressor-like DNA-binding domain, MADS box domain and MIZ were identified in Dictyostelid genomes using the Interpro identifiers IPR010982, IPR002100 and IPR004181, respectively and by Blast searches. Phylogenetic trees were inferred and annotated as in figure S1.

The Lambda-cro/C1 type repressors contain a DNA binding helix-turn-helix domain and associate with DNA as dimers (Ohlendorf et al. 1998). Dictyostelid genomes contain only one conserved lambda-repressor type HLH protein. The two *Ddis* proteins result from a recent partial duplication of chromosome 2 in AX3 derived strains. Expression of these proteins is upregulated by inhibition of protein synthesis induced by cycloheximide (Singleton et al. 1988).

The MADS-box transcription factors are widely used in different eukaryote phyla. Proteins belonging to the MADS family function as dimers. The primary DNA-binding element is formed by an anti-parallel coiled coil of two amphipathic alpha-helices, one from each subunit (Messenguy and Dubois 2003). This family of proteins often interacts with other transcription factors or accessory factors, expanding their range of target genes. Dictyostelia have four deeply conserved MADS-box proteins, three similar to serum response factors (SRF) and the other to myocyte enhancer factor 2 (Mef2). *Ppal* has two additional MADS-box proteins. *Ddis srfA* is required for spore coat formation and organization of the actin cytoskeleton during spore formation (Escalante et al. 2004). Cells lacking *srfB* are defective in cytoskeleton related functions, chemotaxis to cAMP and early gene expression (Galardi-Castilla et al. 2008). Cells defective in *mef2A* show reduced growth and impaired prespore and spore differentiation (Galardi-Castilla et al. 2013).

The Msx-Interacting Zinc finger (MIZ) interacts with homeobox protein Msx2 to increase its DNA binding activity (Wu et al. 1997). A MIZ domain is also present in PIAS, an inhibitor of activated STAT transcription factors (Palvimo 2007) and in the Nse1 and Nse2 subunits of the yeast smc5-6 DNA repair complex (McDonald et al. 2003). Dictyostelia have a conserved PIAS protein, named PisA, which antagonizes the function of *Ddis* STAT1 (Kawata et al. 2011) and a conserved Nse zinc finger protein. *Dpur* has an additional Nse Zn protein and *Ddis* three additional MIZ domain protein that are not conserved.

Figure S14A. Myb DNA binding proteins set 1

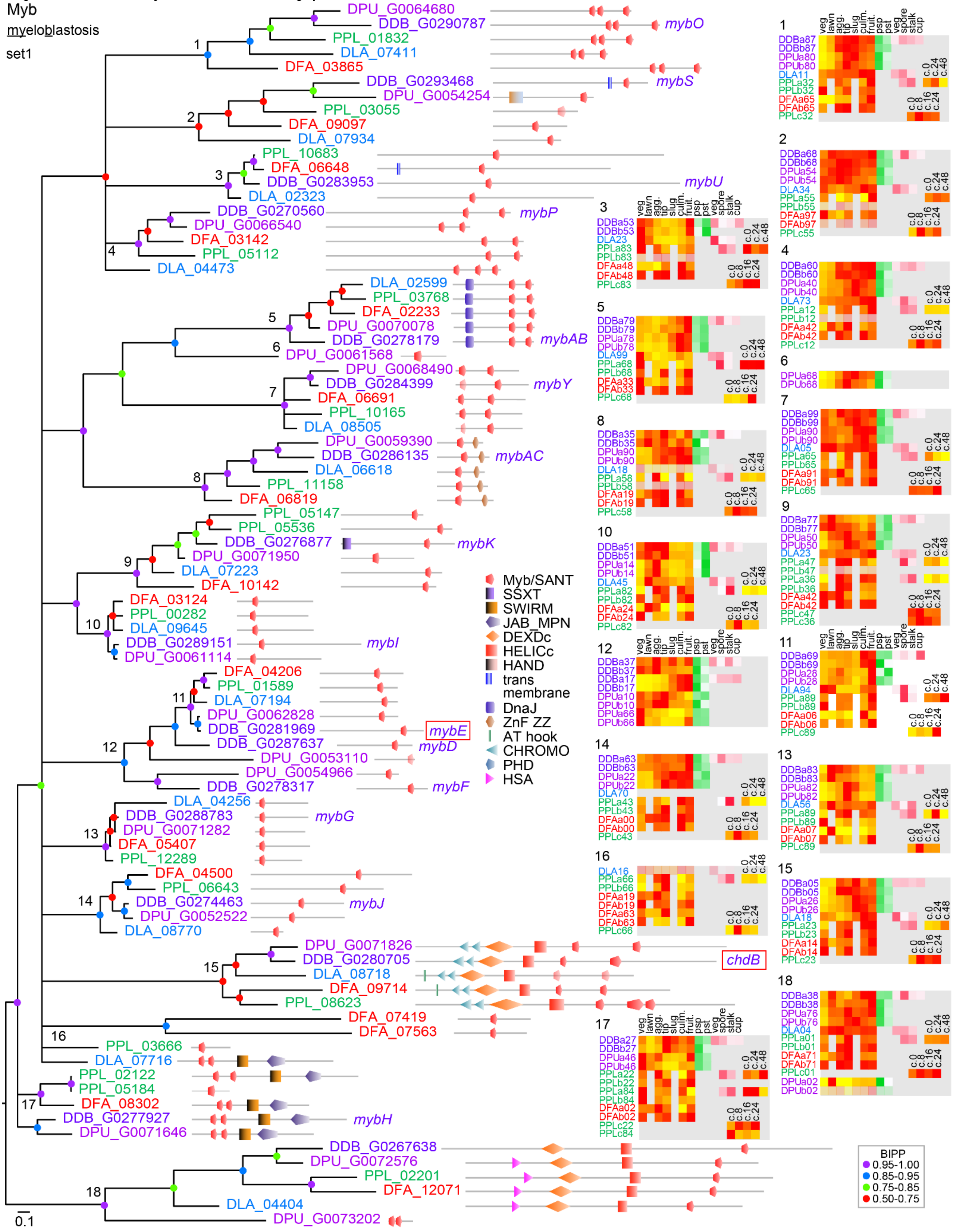

Figure S14B. Myb DNA binding proteins set 2

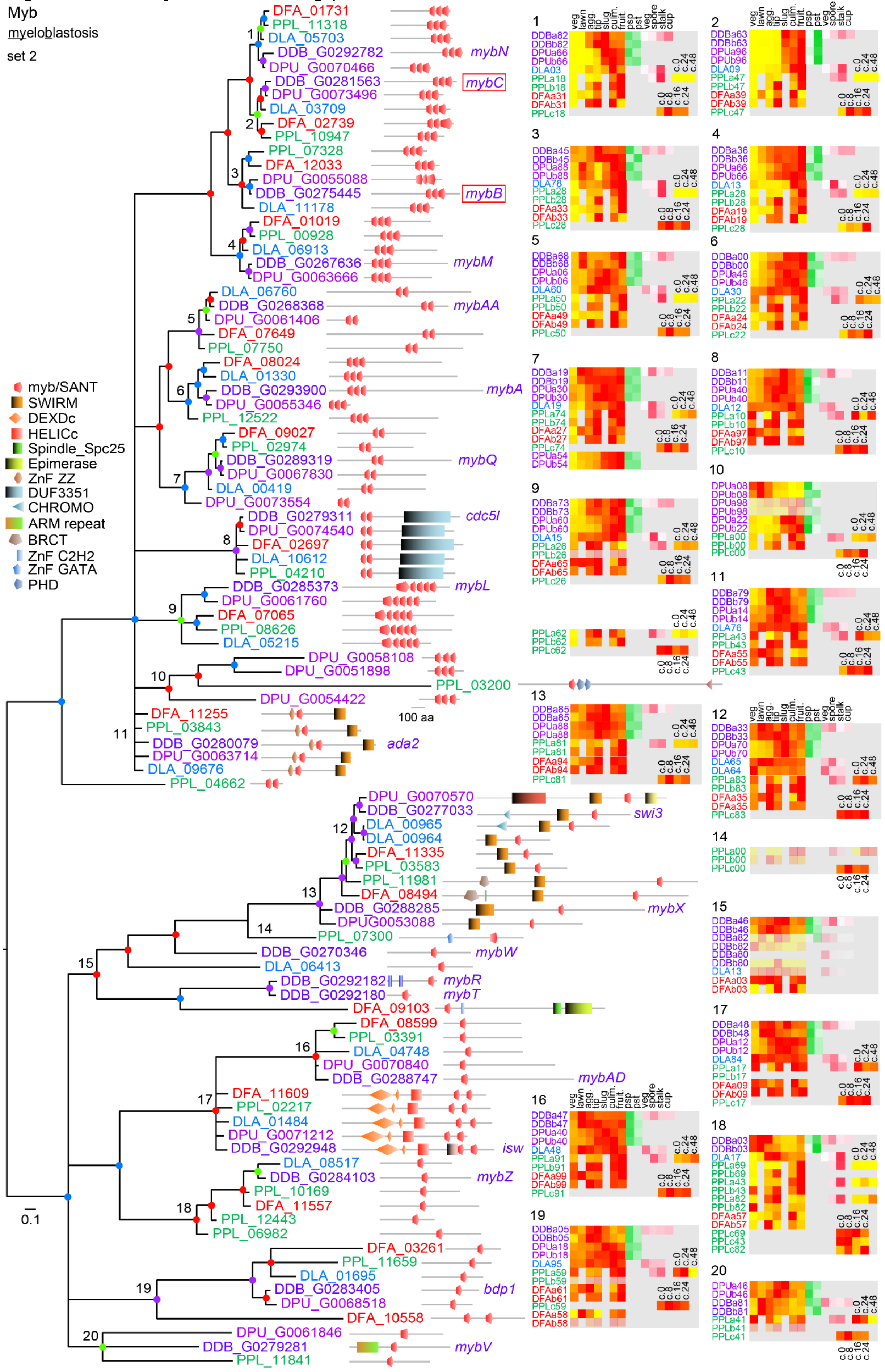

Figure S14. Myb/SANT domain DNA binding proteins

Sequences with Myb domains were retrieved from Dictyostelid genomes with the Interpro Myb domain identifier (IPR017930), which also recovers proteins with the related SANT domain. Dictyostelid proteomes or genomes and were queried further by BlastP or tblastn with the sequences incomplete orthologous sets the *Ddis* mybs. The sequences corresponding to the myb/SANT domains were aligned and a pilot phylogenetic tree was constructed, which showed subdivision into two large branches. Two new trees were constructed from each branch, shown here as sets 1 and 2, and annotated as in figure S1.

The myb domain is named after the retroviral oncogene v-myb (myeloblastosis) and its cellular counterpart c-myb, which both encode DNA-binding proteins (Klempnauer and Sippel 1987; Prouse and Campbell 2012). The highly similar SANT domain is usually part of chromatin remodelling proteins and is considered to interact with histone tails, rather than DNA itself (Aasland et al. 1996; Boyer et al. 2004). Both domains consist of tandem repeats of three alpha-helices that are arranged in a helix-turn-helix motif.

The *Ddis* genome contains 27 proteins that are annotated as mybs A to Z and mybAA, of which mybs A-C,E,G-J,L-Q,S,U,Y and Z are conserved in all taxon groups. We detected three additional mybs: AB, AC and AD that are conserved throughout Dictyostelia. Mybs D and F arose through a group-4 specific duplication of *mybE*, mybX is specific to group 4, but is related to the clade of Swi3 chromatin remodeling proteins. mybR and mybT are *Ddis* specific proteins, while mybV only has a counterpart in *Dpur* and *Ppal*. mybW is not clearly affiliated with any other myb. MybZ is twice duplicated in *Ppal*, while *Ppal*, *Dfas*, *Dlac* and *Dpur* have each a few unique mybs. The different mybs can have from 1 to 5 myb/SANT domains and a variety of other domains that mostly function in either DNA binding or chromatin remodeling. The conserved ada2, chdB, isw, swi2 and swi3 proteins are all homologs of well-established eukaryote chromatin remodeling proteins (Clapier and Cairns 2009), while cdc5l is a component of the eukaryote spliceosome (Burns et al. 1999). Bdp1 is a subunit of TFIIIB, which is required for transcription of tRNA by RNA polIII (Ishiguro et al. 2002).

Developmental roles were previously assigned to the *Ddis* ChdB and mybs B,C and E. MybB is (in addition to CbfA) required for basal expression of adenylate cyclase A (Otsuka and Van Haastert 1998). MybC null mutants show a non-cell autonomous defect in the switch from slug migration to fruiting body formation (Guo et al. 1999). MybE mediates induction of the prestalk gene *ecmA* by DIF-1 (Fukuzawa et al. 2006), and is also required for *ecmB* expression in the lower cup that supports the spore head (Tsujioka et al. 2007). Remarkably, many genes are only DIF-1 inducible in the absence of *mybE* (Yamada et al. 2010). Despite this, similar to DIF-1 less mutants, mybE null mutants form long, weak slugs and fruiting bodies without the basal disc (Saito et al. 2008). The *chdB*-null mutants show delayed mound formation during early development and multiple mis-expressed genes (Platt et al. 2013).

Figure S15. NDT80, NFX1 and Pipsqueak

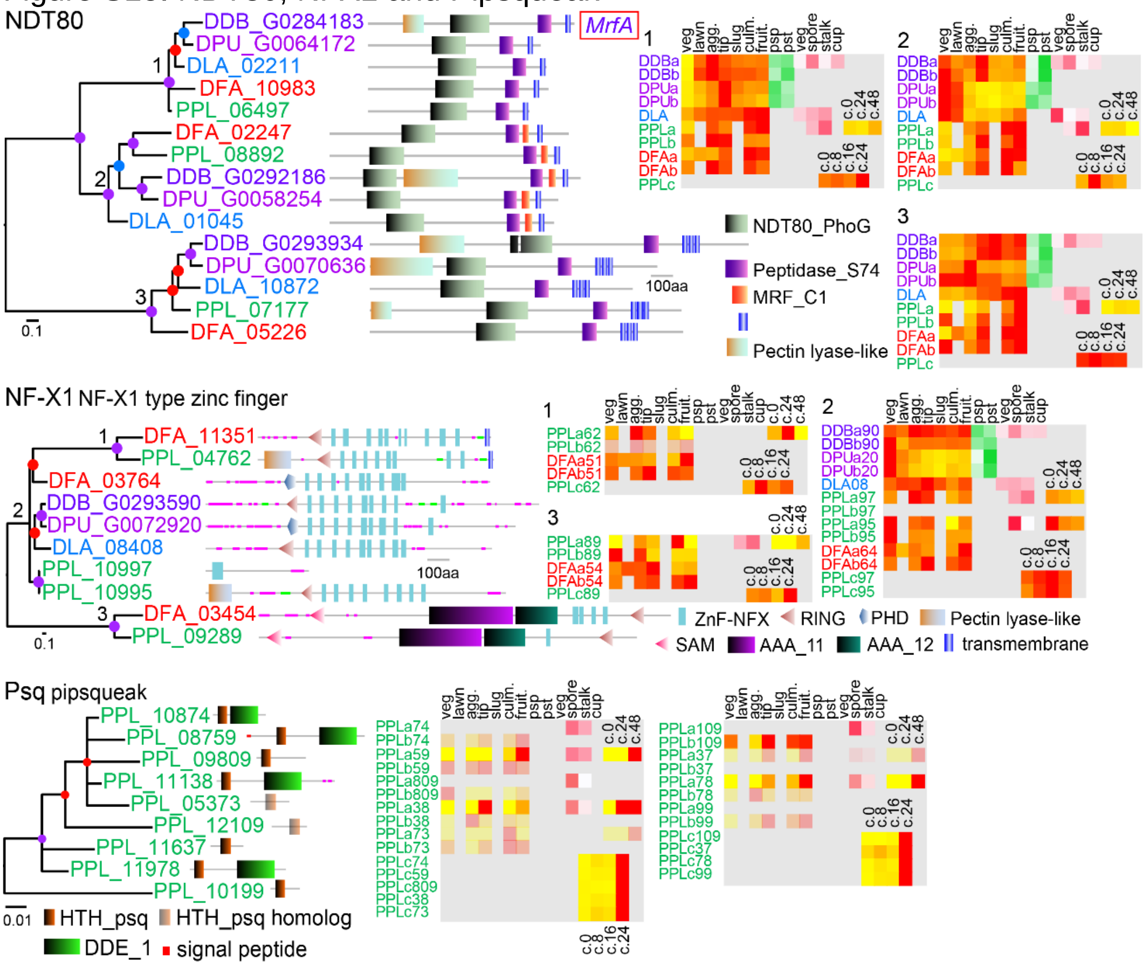

Figure S15. NDT80 and NF-X1 DNA binding proteins

Sequences containing NDT80, NFX1 or Psq-type HTH domains were identified with the Interpro identifier IPR024061 or IPR000967, IPR007889, respectively, and by Blast searches. Phylogenetic trees were inferred and annotated as in figure S1.

The NDT80 DNA-binding domain consists of a core β-sandwich with additional β-sheets and an α-helix (Montano et al. 2002). Transcription factors with this domain are found in Amoebozoa and Opisthokonta. In fungi, NDT80 family proteins are required for regulation of meiosis or nutritional responses (Winter 2012), whereas the metazoan protein, myelin gene regulatory factor (MRF) is a key regulator of CNS myelination (Emery et al. 2009). Interestingly, a human protein, MYRF and a *Ddis* homologue, mrfA are membrane-tethered transcription factors (Li et al. 2013; Senoo et al. 2013). They are released from the endoplasmic reticulum through auto-cleavage by an intramolecular chaperone domain that has similarity to the chaperone of the bacteriophage protein endosialidase. In *Ddis*, the released fragment of mrfA that contains the DNA binding domain translocates to the nucleus, where it acts as an activator of the prestalk gene *ecmA* (Senoo et al. 2012; Senoo et al. 2013). Dictyostelia have three deeply conserved genes with an NDT80 DNA binding domain, including *mrfA*. All three genes have transmembrane domains and the chaperone domain, and are conserved across 4 taxon groups.

The NF-X1 zinc finger with a unique pattern of cysteine and histidines is present in species ranging from plants to humans. The zinc finger domain binds to an X-box motif in the promoter of human major histocompatibility complex class II genes, acting as a transcriptional repressor (Song et al. 1994) In *Arabidopsis*, two NF-X1 proteins antagonistically control expression of stress-related genes (Lisso et al. 2006). Dictyostelia have one deeply conserved gene with tandem NF-X1 repeats, while *Dfas* and *Ppal* have two additional NF-X1 containing genes. None of the genes have been functionally characterized.

Pipsqueak (psq) is a helix-turn-helix type transcription factor with many developmental roles in *Drosophila*, where it binds to GAGAG consensus motifs in target genes (Lehmann et al. 1998). In Dictyostelia, only *Ppal* has a number of *psq* genes with high sequence similarity in the psq motif, which is however not always detected as such. In four *Ppal* genes, the psq domain is combined with a DDE_1 domain. DDE domains generally encode endonucleases required for efficient DNA transposition (Nesmelova and Hackett 2010). The *Ppal psq* genes are mostly poorly expressed in growth and development, but strongly upregulated in late encystation.

Figure S16. STAT, TF2, TMF-1 and WRKY

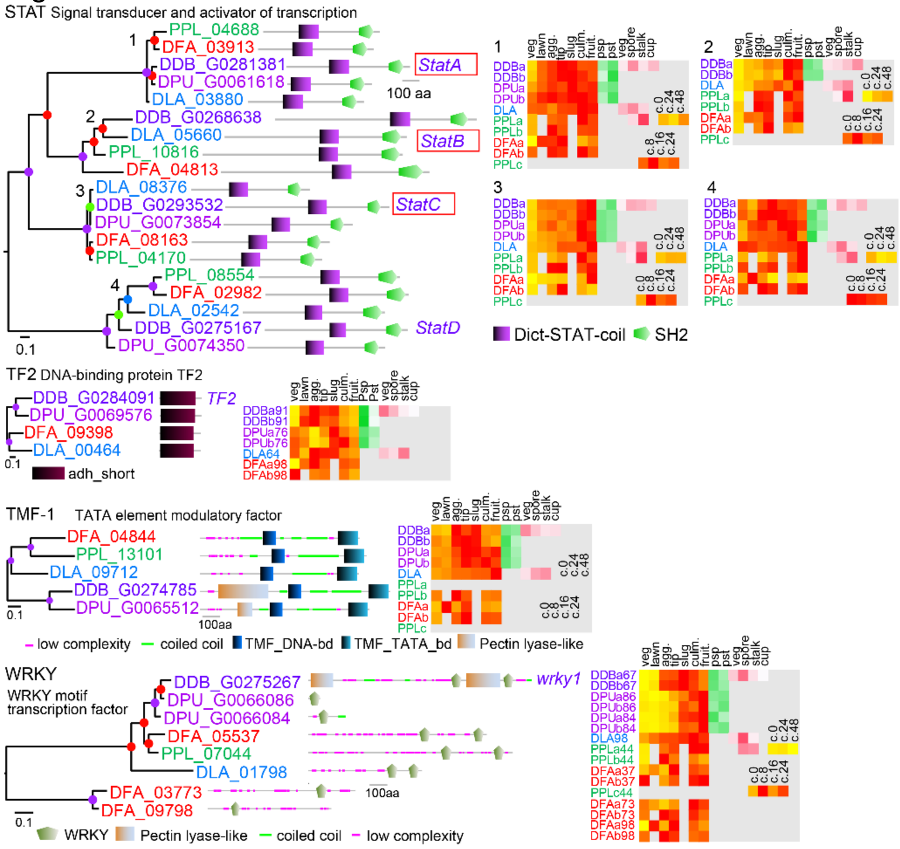

Figure S16. STAT, TF2, TMF-1 and WRKY transcriptional regulators.

Sequences containing STAT, TMF-1 or WRKY domains were identified by the Interpro identifiers IPR015347, IPR022092/IPR022091, or IPR003657, respectively, and by Blast queries. Phylogenetic trees were inferred and annotated as in figure S1.

STATs (Signal Transducer and Activator of Transcription) are widely used metazoan transcription factors with roles in development, proliferation and immune response. In the canonical metazoan pathway STATs are phosphorylated by the tyrosine kinase JAK, which causes their dimerization and accumulation in the nucleus, where they bind to target genes (Darnell 1997). *Ddis* has four STATs a-d. STATa is nuclear translocated in response to cAMP binding to cell surface cAMP receptors (Araki et al. 1998). It is required for efficient chemotaxis and inhibits expression of the stalk gene *ecmB* in the prestalk region of slugs. STATa null mutants also show prolonged slug migration, but eventually form abnormal fruiting structures with few, if any, stalk cells (Mohanty et al. 1999). STATb null mutants gradually disappear when co-cultured with wild-type, but are otherwise normal (Zhukovskaya et al. 2004). StatC is nuclear translocated both in response to hyperosmotic stress and to stimulation with DIF-1 (Araki et al. 2003). STATc null mutants show a minor growth defect, a 1-2 h acceleration of early development and prolonged slug migration (Fukuzawa et al. 2001). No roles for STATd have been uncovered. All four STATs are conserved throughout Dictyostelia, inclusive of their domain architecture.

TF2 was identified in *Ddis* as a protein binding to the 5'C box of the glycogen phosphorylase 2 promoter (Warner and Rutherford 2000). Its short-chain dehydrogenase domain is commonly found in NAD or NADP-dependent oxidoreductases (Jornvall et al. 1995).

TMF-1 (TATA element modulatory factor) functions both as a Golgi protein involved in membrane trafficking (Fridmann-Sirkis et al. 2004; Yamane et al. 2007) and a nuclear protein that competes with TATA binding protein (TBP) for binding to some promoters with the RNA polymerase II TATA box (Garcia et al. 1992). Dictyostelia contain a single well conserved TMF-1 type protein of unknown function.

WRKY transcription factors contain a conserved WRKYGQK motif and C2-H2 or C2-H-C zinc-finger-like motifs and bind to TTGAC(C/T) promoter elements. They are very abundant in plants, where they have diverse biological functions (Bakshi and Oelmuller 2014), but are also found in *Giardia*. WRKY proteins are classified by the number of WRKY domains and features of the zinc-finger. WRKY proteins with two WRKY domains belong to group I, whereas those with one WRKY domain belong to groups II or III. Group I and II share the C2H2 Zn finger motif, while Group III have a C2HC motif. Dictyostelids have a conserved group I WRKY. The two single domain *Dpur* proteins are likely part of the same (mis-annotated) gene. *Dfas* has two additional group II type WRKY proteins.

Figure S17A. General transcription factors set 1

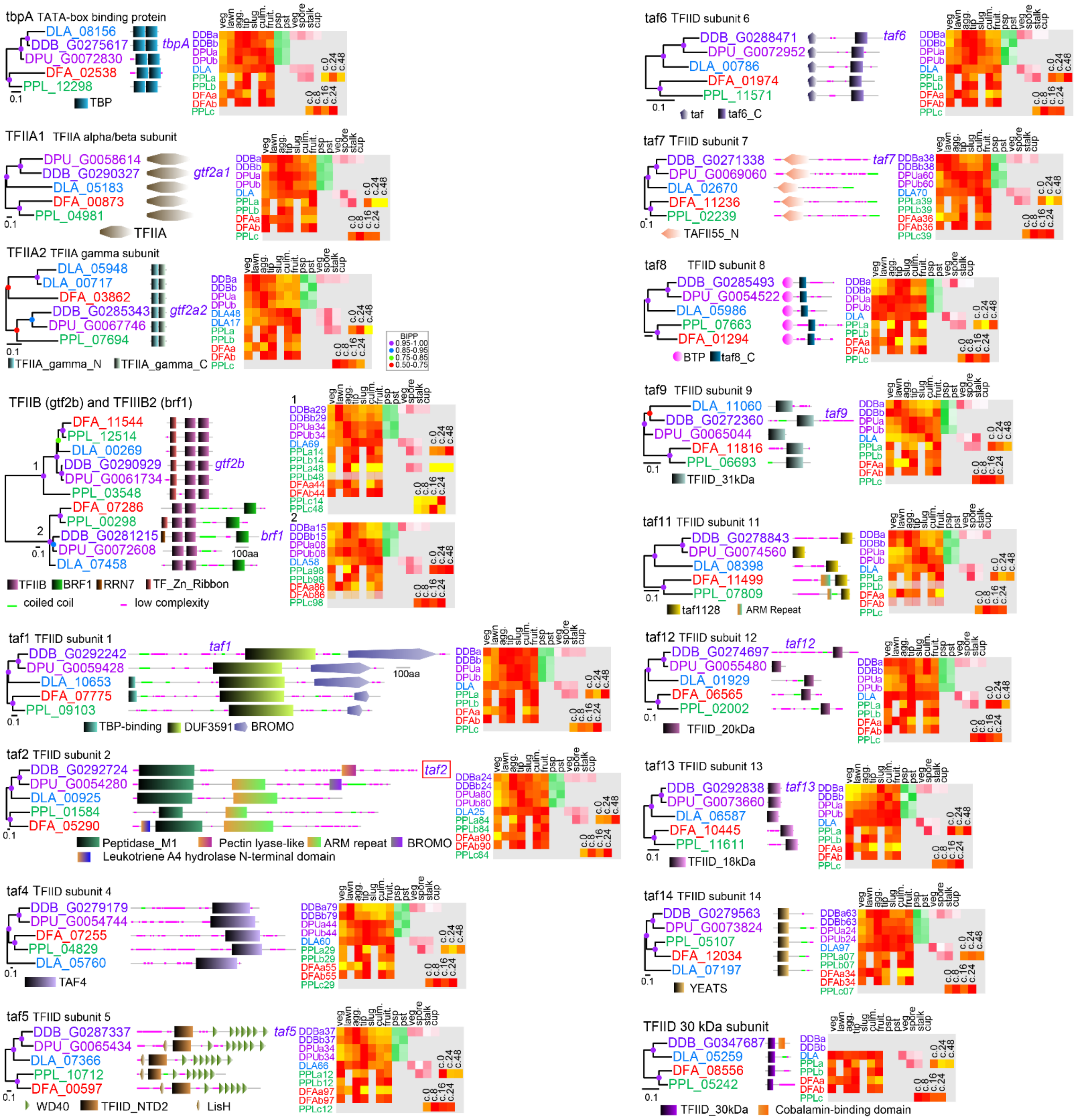

Figure S17B. General transcription factors set 2

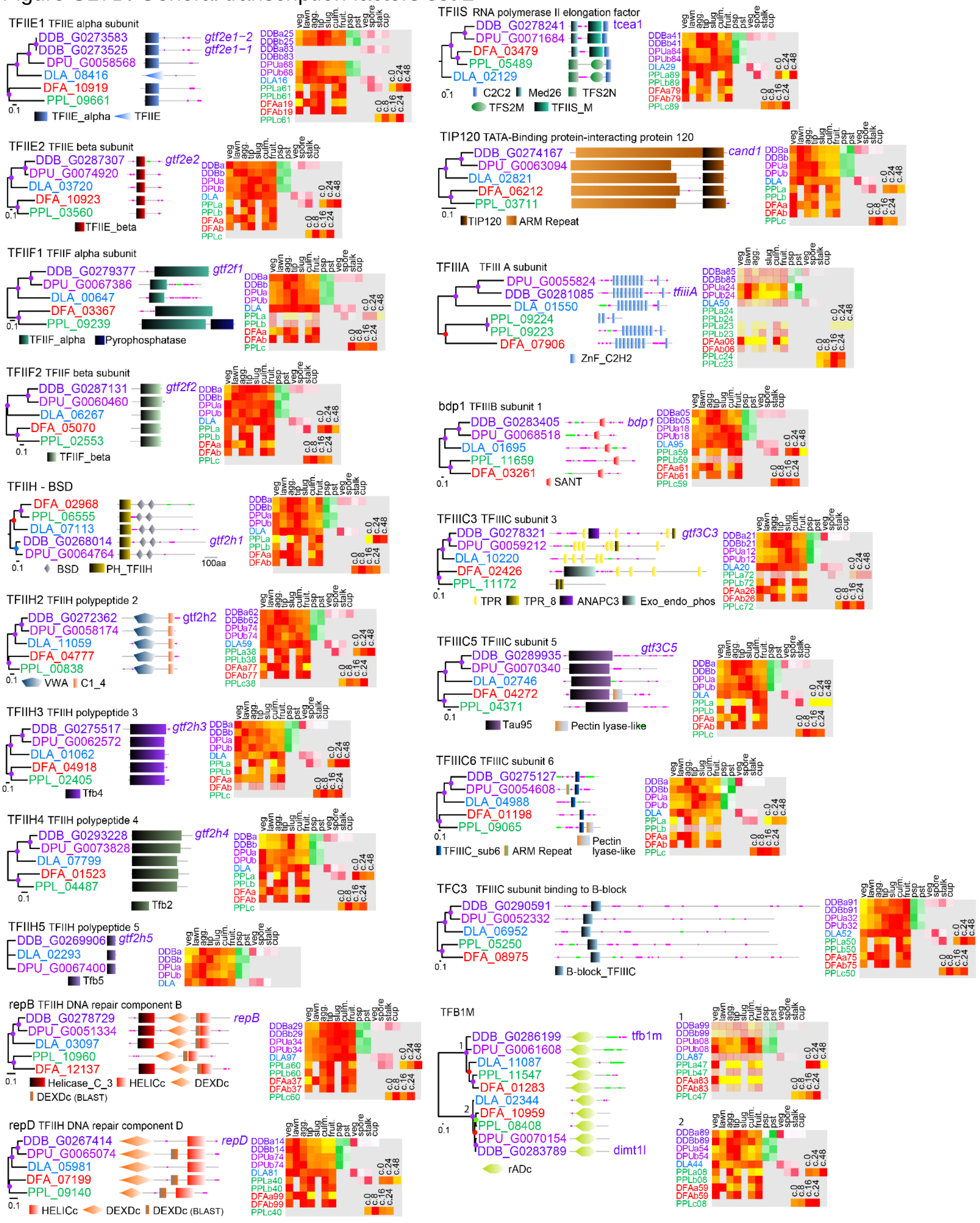

Figure S17. General transcription factors

In addition to sequence-specific transcriptional regulators, eukaryote genes require one of three general transcription factor complexes to initiate transcription. These complexes incorporate the TATA-box binding protein (TBP), any of the three RNA polymerases, RNA pol. I, II and III and up to 14 other proteins (Cooper

and Hausman 2016). Protein-coding genes are transcribed by RNA pol. II, large ribosomal RNAs are transcribed by RNA pol. I and small ribosomal RNAs and transfer RNAs are transcribed by RNA pol. III. Formation of the RNA pol. II transcription complex initiates with the binding of TBP and TBP-associated factors (TAFs) to the TATAA sequence that resides 25-30 nt upstream of the transcription start site. This complex, called TFIID, then sequentially binds TFIIB, RNA pol. II and TFIIF, TFIIE and TFIIH. Several other factors can additionally be recruited to this large complex. For RNA pol I. mediated transcription, TBP associates with other proteins to form SL1, which binds together with the transcription factor UBF to promoters of the large ribosomal RNAs and then recruits RNA pol. I to initiate transcription. RNA pol. III transcription of transfer RNAs initiates by binding of TFIIIC downstream of the transcription start site followed by TFIIIB and RNA pol. III. Transcription of the 5S rRNA additionally requires TFIIIA (Cooper and Hausman 2016).

Most of the proteins required for RNA pol. II and pol III mediated transcription were annotated to the *Ddis* genome in Dictybase (Basu et al. 2015) and blastP searches showed them to be conserved across Dictyostelia. In addition, searches with metazoan sequences revealed the presence of TAFs 4, 8, 11 and 14 across Dictyostelia, only TAF3 was not detected. Also neither of the RNA pol. I associated TFs could be found in Dictyostelia.

Figure S18. Phylogeny-wide change in general transcription factors

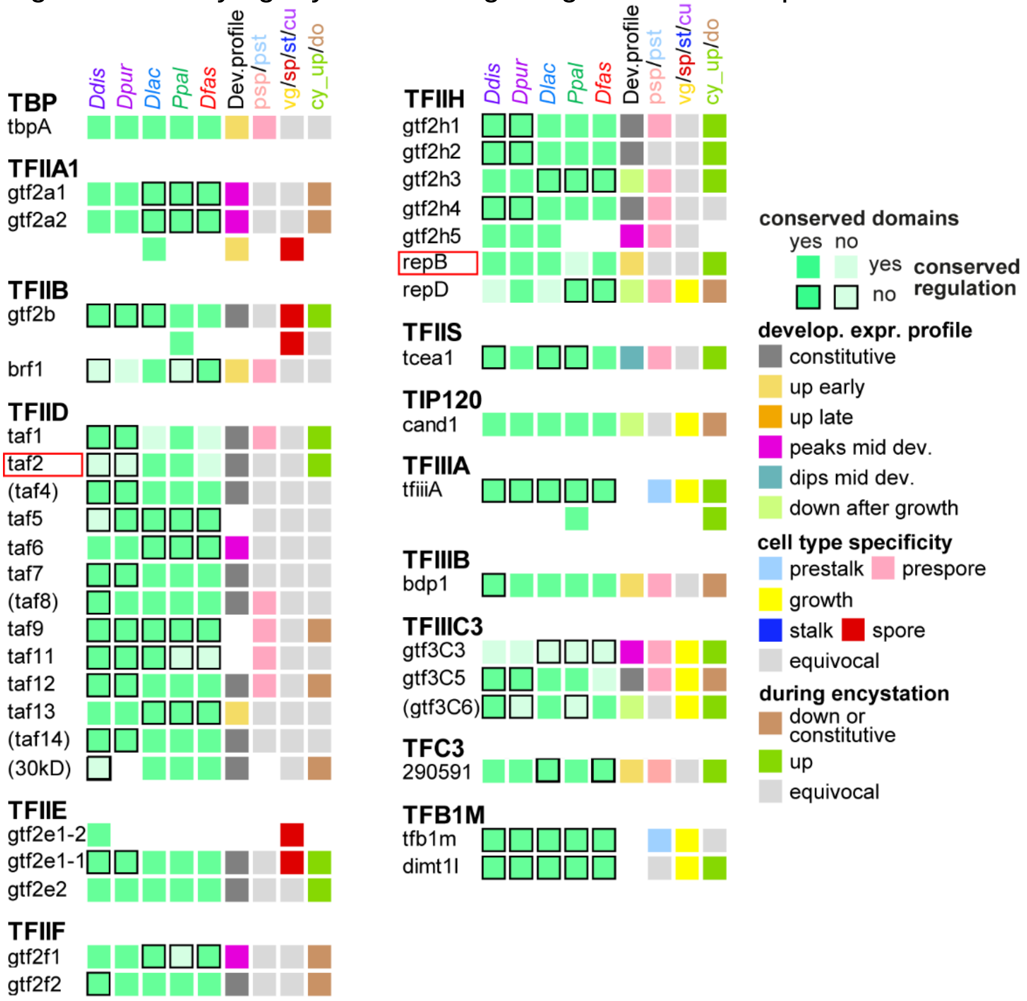

Figure S18. Phylogeny-wide change in general transcription factors
Summary data on orthology and conservation of functional domains, developmental regulation and cell type specificity of components of the general transcription initiation complexes II and III. See the legend to figure 2 for explanation of the colour coding of feature states. Prepared from analyses presented in Figure S17 and summarized in Supplemental_Table_S2. Gene names in parentheses are not yet annotated in Dictybase (Basu et al. 2015).

Supplementary references

Aasland R, Stewart AF, Gibson T. 1996. The SANT domain: a putative DNA-binding domain in the SWI-SNF and ADA complexes, the transcriptional co-repressor N-CoR and TFIIIB. *Trends Biochem Sci* **21**: 87-88.

Andersen SU, Algreen-Petersen RG, Hoedl M, Jurkiewicz A, Cvitanich C, Braunschweig U, Schauser L, Oh SA, Twell D, Jensen EO. 2007. The conserved cysteine-rich domain of a tesmin/TSO1-like protein binds zinc in vitro and TSO1 is required for both male and female fertility in Arabidopsis thaliana. *Journal of experimental botany* **58**: 3657-3670.

Araki T, Gamper M, Early A, Fukuzawa M, Abe T, Kawata T, Kim E, Firtel RA, Williams JG. 1998. Developmentally and spatially regulated activation of a Dictyostelium STAT protein by a serpentine receptor. *EMBO J* **17**: 4018-4028.

Araki T, Tsujioka M, Abe T, Fukuzawa M, Meima M, Schaap P, Morio T, Urushihara H, Katoh M, Maeda M et al. 2003. A STAT-regulated, stress-induced signalling pathway in Dictyostelium. *J Cell Sci* **116**: 2907-2915.

Bakshi M, Oelmuller R. 2014. WRKY transcription factors: Jack of many trades in plants. *Plant signaling & behavior* **9**: e27700.

Basu S, Fey P, Jimenez-Morales D, Dodson RJ, Chisholm RL. 2015. dictyBase 2015: Expanding data and annotations in a new software environment. *genesis* **53**: 523-534.

Bolognese F, Imbriano C, Caretti G, Mantovani R. 2000. Cloning and characterization of the histone-fold proteins YBL1 and YCL1. *Nucleic acids research* **28**: 3830-3838.

Boyer LA, Latek RR, Peterson CL. 2004. The SANT domain: a unique histone-tail-binding module? *Nat Rev Mol Cell Biol* **5**: 158-163.

Brown JM, Firtel RA. 2001. Functional and regulatory analysis of the dictyostelium G-box binding factor. In *Dev Biol*, Vol 234, pp. 521-534.

Bürglin TR, Affolter M. 2016. Homeodomain proteins: an update. *Chromosoma* **125**: 497-521.

Burns CG, Ohi R, Krainer AR, Gould KL. 1999. Evidence that Myb-related CDC5 proteins are required for pre-mRNA splicing. *Proc Natl Acad Sci U S A* **96**: 13789-13794.

Cai H, Katoh-Kurasawa M, Muramoto T, Santhanam B, Long Y, Li L, Ueda M, Iglesias PA, Shaulsky G, Devreotes PN. 2014. Nucleocytoplasmic shuttling of a GATA transcription factor functions as a development timer. *Science* **343**: 1249531.

Cai Z, Wang Y, Yu W, Xiao J, Li Y, Liu L, Zhu C, Tan K, Deng Y, Yuan W et al. 2006. hnulp1, a basic helix-loop-helix protein with a novel transcriptional repressive domain, inhibits transcriptional activity of serum response factor. *Biochem Biophys Res Commun* **343**: 973-981.

Chang W-T, Newell PC, Gross JD. 1996. Identification of the cell fate gene Stalky in Dictyostelium. *Cell* **87**: 471-481.

Clapier CR, Cairns BR. 2009. The Biology of Chromatin Remodeling Complexes. *Annual Review of Biochemistry* **78**: 273-304.

Clissold PM, Ponting CP. 2001. JmjC: cupin metalloenzyme-like domains in jumonji, hairless and phospholipase A2beta. *Trends in biochemical sciences* **26**: 7-9.

Clos J, Westwood JT, Becker PB, Wilson S, Lambert K, Wu C. 1990. Molecular cloning and expression of a hexameric Drosophila heat shock factor subject to negative regulation. *Cell* **63**: 1085-1097.

Cooper J, Hausman R. 2016. *The Cell: A Molecular Approach*. Oxford University Press, Oxford, UK.

Darnell JE, Jr. 1997. STATs and gene regulation. *Science* **277**: 1630-1635.

Diao Y, Guo X, Li Y, Sun K, Lu L, Jiang L, Fu X, Zhu H, Sun H, Wang H et al. 2012. Pax3/7BP is a Pax7- and Pax3-binding protein that regulates the proliferation of muscle precursor cells by an epigenetic mechanism. *Cell Stem Cell* **11**: 231-241.

Emery B, Agalliu D, Cahoy JD, Watkins TA, Dugas JC, Mulinyawe SB, Ibrahim A, Ligon KL, Rowitch DH, Barres BA. 2009. Myelin gene regulatory factor is a critical transcriptional regulator required for CNS myelination. *Cell* **138**: 172-185.

Escalante R, Yamada Y, Cotter D, Sastre L, Sameshima M. 2004. The MADS-box transcription factor SrfA is required for actin cytoskeleton organization and spore coat stability during Dictyostelium sporulation. *Mech Dev* **121**: 51-56.

Fanciulli M, Bruno T, Di Padova M, De Angelis R, Iezzi S, Iacobini C, Floridi A, Passananti C. 2000. Identification of a novel partner of RNA polymerase II subunit 11, Che-1, which interacts with and affects the growth suppression function of Rb. *FASEB journal : official publication of the Federation of American Societies for Experimental Biology* **14**: 904-912.

Fridmann-Sirkis Y, Siniossoglou S, Pelham HR. 2004. TMF is a golgin that binds Rab6 and influences Golgi morphology. *BMC cell biology* **5**: 18.

Fukuzawa M, Araki T, Adrian I, Williams JG. 2001. Tyrosine phosphorylation-independent nuclear translocation of a *Dictyostelium* STAT in response to DIF signaling. *Mol Cell* **7**: 779-788.

Fukuzawa M, Hopper N, Williams J. 1997. cudA: a *Dictyostelium* gene with pleiotropic effects on cellular differentiation and slug behaviour. *Development* **124**: 2719-2728.

Fukuzawa M, Zhukovskaya NV, Yamada Y, Araki T, Williams JG. 2006. Regulation of Dictyostelium prestalk-specific gene expression by a SHAQKY family MYB transcription factor. *Development* **133**: 1715-1724.

Galardi-Castilla M, Fernandez-Aguado I, Suarez T, Sastre L. 2013. Mef2A, a homologue of animal Mef2 transcription factors, regulates cell differentiation in Dictyostelium discoideum. *BMC Dev Biol* **13**: 12.

Galardi-Castilla M, Pergolizzi B, Bloomfield G, Skelton J, Ivens A, Kay RR, Bozzaro S, Sastre L. 2008. SrfB, a member of the Serum Response Factor family of transcription factors, regulates starvation response and early development in Dictyostelium. *Dev Biol* **316**: 260-274.

Garcia JA, Ou SH, Wu F, Lusis AJ, Sparkes RS, Gaynor RB. 1992. Cloning and chromosomal mapping of a human immunodeficiency virus 1 "TATA" element modulatory factor. *Proceedings of the National Academy of Sciences* **89**: 9372-9376.

Georgieva S, Nabirochkina E, Dilworth FJ, Eickhoff H, Becker P, Tora L, Georgiev P, Soldatov A. 2001. The novel transcription factor e(y)2 interacts with TAF(II)40 and potentiates transcription activation on chromatin templates. *Molecular and cellular biology* **21**: 5223-5231.

Gloeckner G, Lawal HM, Felder M, Singh R, Singer G, Weijer CJ, Schaap P. 2016. The multicellularity genes of dictyostelid social amoebas. *Nature communications* **7**: 12085.

Grzenda A, Lomberk G, Zhang JS, Urrutia R. 2009. Sin3: master scaffold and transcriptional corepressor. *Biochimica et biophysica acta* **1789**: 443-450.

Guo K, Anjard C, Harwood A, Kim HJ, Newell PC, Gross JD. 1999. A myb-related protein required for culmination in Dictyostelium. *Development* **126**: 2813-2822.

Hai T, Curran T. 1991. Cross-family dimerization of transcription factors Fos/Jun and ATF/CREB alters DNA binding specificity. *Proc Natl Acad Sci U S A* **88**: 3720-3724.

Han Z, Firtel RA. 1998. The homeobox-containing gene wariai regulates anterior-posterior patterning and cell-type homeostasis in dictyostelium. *Development* **125**: 313-325.

Harrison MM, Ceol CJ, Lu X, Horvitz HR. 2006. Some C. elegans class B synthetic multivulva proteins encode a conserved LIN-35 Rb-containing complex distinct from a NuRD-like complex. *Proc Natl Acad Sci U S A* **103**: 16782-16787.

Herrscher RF, Kaplan MH, Lelsz DL, Das C, Scheuermann R, Tucker PW. 1995. The immunoglobulin heavy-chain matrix-associating regions are bound by Bright: a B cell-specific trans-activator that describes a new DNA-binding protein family. *Genes & development* **9**: 3067-3082.

Hjorth AL, Khanna NC, Firtel RA. 1989. A trans-acting factor required for cAMP-induced gene expression in Dictyostelium is regulated developmentally and induced by cAMP. *Genes Dev* **3**: 747-759.

Hjorth AL, Pears C, Williams JG, Firtel RA. 1990. A developmentally regulated trans-acting factor recognizes dissimilar G/C-rich elements controlling a class of cAMP- inducible Dictyostelium genes. *Genes Dev* **4**: 419-432.

Horn J, DietzSchmidt A, Zundorf I, Garin L, Dingermann T, Winckler T. 1999. A Dictyostelium protein binds to distinct oligo(dA)center dot oligo(dT) DNA sequences in the C-module of the retrotransposable element DRE. *European Journal of Biochemistry* **265**: 441-448.

Huang E, Talukder S, Hughes TR, Curk T, Zupan B, Shaulsky G, Katoh-Kurasawa M. 2011. BzpF is a CREB-like transcription factor that regulates spore maturation and stability in Dictyostelium. *Developmental Biology* **358**: 137-146.

Huang EY, Blagg SL, Keller T, Katoh M, Shaulsky G, Thompson CRL. 2006. bZIP transcription factor interactions regulate DIF responses in Dictyostelium. *Development* **133**: 449-458.

Ishiguro A, Kassavetis GA, Geiduschek EP. 2002. Essential roles of Bdp1, a subunit of RNA polymerase III initiation factor TFIIIB, in transcription and tRNA processing. *Molecular and cellular biology* **22**: 3264-3275.

Iuchi S. 2001. Three classes of C2H2 zinc finger proteins. *Cellular and molecular life sciences : CMLS* **58**: 625-635.

Iwahara J, Iwahara M, Daughdrill GW, Ford J, Clubb RT. 2002. The structure of the Dead ringer-DNA complex reveals how AT-rich interaction domains (ARIDs) recognize DNA. *The EMBO journal* **21**: 1197-1209.

Jones S. 2004. An overview of the basic helix-loop-helix proteins. *Genome Biology* **5**: 226.

Jornvall H, Persson B, Krook M, Atrian S, Gonzalez-Duarte R, Jeffery J, Ghosh D. 1995. Short-chain dehydrogenases/reductases (SDR). *Biochemistry* **34**: 6003-6013.

Kageyama R, Pastan I. 1989. Molecular cloning and characterization of a human DNA binding factor that represses transcription. *Cell* **59**: 815-825.

Katoh-Kurasawa M, Santhanam B, Shaulsky G. 2016. The GATA transcription factor gene gtaG is required for terminal differentiation in Dictyostelium. *J Cell Sci* doi:10.1242/jcs.181545.

Kawata T, Hirano T, Ogasawara S, Aoshima R, Yachi A. 2011. Evidence for a functional link between Dd-STATa and Dd-PIAS, a Dictyostelium PIAS homologue. *Dev Growth Differ* **53**: 897-909.

Keller T, Thompson CR. 2008. Cell type specificity of a diffusible inducer is determined by a GATA family transcription factor. *Development* **135**: 1635-1645.

Kibler K, Nguyen TL, Svetz J, Van Driessche N, Ibarra M, Thompson C, Shaw C, Shaulsky G. 2003. A novel developmental mechanism in Dictyostelium revealed in a screen for communication mutants. *Dev Biol* **259**: 193-208.

Kim JS, Seo JH, Yim HS, Kang SO. 2011. Homeoprotein Hbx4 represses the expression of the adhesion molecule DdCAD-1 governing cytokinesis and development. *FEBS Lett* **585**: 1864-1872.

Kin K, Forbes G, Cassidy A, Schaap P. 2018. Cell-type specific RNA-Seq reveals novel roles and regulatory programs for terminally differentiated Dictyostelium cells. *BMC Genomics* **19**: 764.

Klempnauer KH, Sippel AE. 1987. The highly conserved amino-terminal region of the protein encoded by the v-myb oncogene functions as a DNA-binding domain. *The EMBO journal* **6**: 2719-2725.

Lee JW, Choi HS, Gyuris J, Brent R, Moore DD. 1995. Two classes of proteins dependent on either the presence or absence of thyroid hormone for interaction with the thyroid hormone receptor. *Molecular endocrinology (Baltimore, Md)* **9**: 243-254.

Lehmann M, Siegmund T, Lintermann KG, Korge G. 1998. The pipsqueak protein of Drosophila melanogaster binds to GAGA sequences through a novel DNA-binding domain. *The Journal of biological chemistry* **273**: 28504-28509.

Li Z, Park Y, Marcotte EM. 2013. A Bacteriophage tailspike domain promotes self-cleavage of a human membrane-bound transcription factor, the myelin regulatory factor MYRF. *PLoS Biol* **11**: e1001624.

Lin R, Teng Y, Park H-J, Ding L, Black C, Fang P, Wang H. 2008. Discrete and Essential Roles of the Multiple Domains of Arabidopsis FHY3 in Mediating Phytochrome A Signal Transduction. *Plant Physiology* **148**: 981-992.

Lisso J, Altmann T, Mussig C. 2006. The AtNFXL1 gene encodes a NF-X1 type zinc finger protein required for growth under salt stress. *FEBS letters* **580**: 4851-4856.

MacPherson S, Larochelle M, Turcotte B. 2006. A Fungal Family of Transcriptional Regulators: the Zinc Cluster Proteins. *Microbiology and Molecular Biology Reviews* **70**: 583-604.

Mantovani R. 1999. The molecular biology of the CCAAT-binding factor NF-Y. *Gene* **239**: 15-27.

McDonald WH, Pavlova Y, Yates JR, 3rd, Boddy MN. 2003. Novel essential DNA repair proteins Nse1 and Nse2 are subunits of the fission yeast Smc5-Smc6 complex. *The Journal of biological chemistry* **278**: 45460-45467.

Mermelstein F, Yeung K, Cao J, Inostroza JA, Erdjument-Bromage H, Eagelson K, Landsman D, Levitt P, Tempst P, Reinberg D. 1996. Requirement of a corepressor for Dr1-mediated repression of transcription. *Genes & development* **10**: 1033-1048.

Messenguy F, Dubois E. 2003. Role of MADS box proteins and their cofactors in combinatorial control of gene expression and cell development. *Gene* **316**: 1-21.

Miller J, McLachlan AD, Klug A. 1985. Repetitive zinc-binding domains in the protein transcription factor IIIA from Xenopus oocytes. *The EMBO journal* **4**: 1609-1614.

Mishra H, Bhadoriya P, Saran S. 2017. Disruption of homeobox containing gene, hbx9 results in the deregulation of prestalk cell patterning in Dictyostelium discoideum. *Differentiation* **94**: 27-36.

Mohanty S, Jermyn KA, Early A, Kawata T, Aubry L, Ceccarelli A, Schaap P, Williams JG, Firtel RA. 1999. Evidence that the Dictyostelium Dd-STATa protein is a repressor that regulates commitment to stalk cell differentiation and is also required for efficient chemotaxis. *Development* **126**: 3391-3405.

Montano SP, Cote ML, Fingerman I, Pierce M, Vershon AK, Georgiadis MM. 2002. Crystal structure of the DNA-binding domain from Ndt80, a transcriptional activator required for meiosis in yeast. *Proc Natl Acad Sci U S A* **99**: 14041-14046.

Mu X, Spanos SA, Shiloach J, Kimmel A. 2001. CRTF is a novel transcription factor that regulates multiple stages of Dictyostelium development. *Development* **128**: 2569-2579.

Nesmelova IV, Hackett PB. 2010. DDE transposases: Structural similarity and diversity. *Advanced drug delivery reviews* **62**: 1187-1195.

Nieto-Sotelo J, Ichida A, Quail PH. 1994. PF1: an A-T hook-containing DNA binding protein from rice that interacts with a functionally defined d(AT)-rich element in the oat phytochrome A3 gene promoter. *The Plant cell* **6**: 287-301.

Ohlendorf DH, Tronrud DE, Matthews BW. 1998. Refined structure of Cro repressor protein from bacteriophage lambda suggests both flexibility and plasticity. *Journal of molecular biology* **280**: 129-136.

Omichinski J, Clore G, Schaad O, Felsenfeld G, Trainor C, Appella E, Stahl S, Gronenborn A. 1993. NMR structure of a specific DNA complex of Zn-containing DNA binding domain of GATA-1. *Science* **261**: 438-446.

Otsuka H, Van Haastert PJM. 1998. A novel myb homolog initiates *Dictyostelium* development by induction of adenylyl cyclase expression. *Genes & Dev* **12**: 1738-1748.

Palvimo JJ. 2007. PIAS proteins as regulators of small ubiquitin-related modifier (SUMO) modifications and transcription. *Biochemical Society transactions* **35**: 1405-1408.

Pan T, Coleman JE. 1990. GAL4 transcription factor is not a "zinc finger" but forms a Zn(II)2Cys6 binuclear cluster. *Proc Natl Acad Sci U S A* **87**: 2077-2081.

Parikh A, Huang E, Dinh C, Zupan B, Kuspa A, Subramanian D, Shaulsky G. 2010a. New components of the Dictyostelium PKA pathway revealed by Bayesian analysis of expression data. *BMC Bioinformatics* **11**: 163.

Parikh A, Miranda ER, Katoh-Kurasawa M, Fuller D, Rot G, Zagar L, Curk T, Sucgang R, Chen R, Zupan B et al. 2010b. Conserved developmental transcriptomes in evolutionarily divergent species. *Genome Biol* **11**: R35.

Parkinson K, Buttery NJ, Wolf JB, Thompson CR. 2011. A simple mechanism for complex social behavior. *PLoS Biol* **9**: e1001039.

Pascual-García P, Govind CK, Queralt E, Cuenca-Bono B, Llopis A, Chavez S, Hinnebusch AG, Rodríguez-Navarro S. 2008. Sus1 is recruited to coding regions and functions during transcription elongation in association with SAGA and TREX2. *Genes & development* **22**: 2811-2822.

Phillips JE, Huang E, Shaulsky G, Gomer RH. 2011. The putative bZIP transcription factor BzpN slows proliferation and functions in the regulation of cell density by autocrine signals in Dictyostelium. *PLoS ONE* **6**: e21765.

Platt JL, Rogers BJ, Rogers KC, Harwood AJ, Kimmel AR. 2013. Different CHD chromatin remodelers are required for expression of distinct gene sets and specific stages during development of Dictyostelium discoideum. *Development* **140**: 4926-4936.

Prouse MB, Campbell MM. 2012. The interaction between MYB proteins and their target DNA binding sites. *Biochimica et biophysica acta* **1819**: 67-77.

Reeves R, Beckerbauer L. 2001. HMGI/Y proteins: flexible regulators of transcription and chromatin structure. *Biochimica et biophysica acta* **1519**: 13-29.

Ronquist F, Huelsenbeck JP. 2003. MrBayes 3: Bayesian phylogenetic inference under mixed models. *Bioinformatics* **19**: 1572-1574.

Saito T, Kato A, Kay RR. 2008. DIF-1 induces the basal disc of the *Dictyostelium* fruiting body. *Dev Biol* **317**: 444-453.

Santhanam B, Cai H, Devreotes PN, Shaulsky G, Katoh-Kurasawa M. 2015. The GATA transcription factor GtaC regulates early developmental gene expression dynamics in Dictyostelium. *Nature communications* **6**: 7551.

Schmith A, Groth M, Ratka J, Gatz S, Spaller T, Siol O, Glockner G, Winckler T. 2013. Conserved gene regulatory function of the carboxy-terminal domain of dictyostelid C-module-binding factor. *Eukaryot Cell* **12**: 460-468.

Schnitzler GR, Fischer WH, Firtel RA. 1994. Cloning and characterization of the G-box binding factor, an essential component of the developmental switch between early and late development in Dictyostelium. *Genes Dev* **8**: 502-514.

Schotta G, Ebert A, Reuter G. 2003. SU(VAR)3-9 is a conserved key function in heterochromatic gene silencing. *Genetica* **117**: 149-158.

Schultz J, Milpetz F, Bork P, Ponting CP. 1998. SMART, a simple modular architecture research tool: identification of signaling domains. *Proc Natl Acad Sci USA* **95**: 5857-5864.

Senoo H, Araki T, Fukuzawa M, Williams JG. 2013. A new kind of membrane-tethered eukaryotic transcription factor that shares an auto-proteolytic processing mechanism with bacteriophage tail-spike proteins. *J Cell Sci* **126**: 5247-5258.

Senoo H, Wang HY, Araki T, Williams JG, Fukuzawa M. 2012. An orthologue of the Myelin-gene Regulatory Transcription Factor regulates Dictyostelium prestalk differentiation. *The International journal of developmental biology* **56**: 325-332.

Sievers F, Higgins DG. 2014. Clustal omega, accurate alignment of very large numbers of sequences. *Methods in molecular biology* **1079**: 105-116.

Singh M, D'Silva L, Holak TA. 2006. DNA-binding properties of the recombinant high-mobility-group-like AT-hook-containing region from human BRG1 protein. *Biological chemistry* **387**: 1469-1478.

Singleton CK, Manning SS, Feng Y. 1988. Effect of protein synthesis inhibition on gene expression during early development of Dictyostelium discoideum. *MolCellBiol* **8**: 10-16.

Siol O, Dingermann T, Winckler T. 2006. The C-module DNA-binding factor mediates expression of the dictyostelium aggregation-specific adenylyl cyclase ACA. *Eukaryot Cell* **5**: 658-664.

Song Z, Krishna S, Thanos D, Strominger JL, Ono SJ. 1994. A novel cysteine-rich sequence-specific DNA-binding protein interacts with the conserved X-box motif of the human major histocompatibility complex class II genes via a repeated Cys-His domain and functions as a transcriptional repressor. *The Journal of experimental medicine* **180**: 1763-1774.

Stros M, Launholt D, Grasser KD. 2007. The HMG-box: a versatile protein domain occurring in a wide variety of DNA-binding proteins. *Cellular and molecular life sciences : CMLS* **64**: 2590-2606.

Sugihara T, Wadhwa R, Kaul SC, Mitsui Y. 1999. A novel testis-specific metallothionein-like protein, tesmin, is an early marker of male germ cell differentiation. *Genomics* **57**: 130-136.

Takeuchi T, Watanabe Y, Takano-Shimizu T, Kondo S. 2006. Roles of jumonji and jumonji family genes in chromatin regulation and development. *Dev Dyn* **235**: 2449-2459.

Thewes S, Krohn S, Schmith A, Herzog S, Stach T, Weissenmayer B, Mutzel R. 2012. The calcineurin dependent transcription factor TacA is involved in development and the stress response of Dictyostelium discoideum. *Eur J Cell Biol* **91**: 789-799.

Trewick SC, McLaughlin PJ, Allshire RC. 2005. Methylation: lost in hydroxylation? *EMBO reports* **6**: 315-320.

Tsujioka M, Zhukovskaya N, Yamada Y, Fukuzawa M, Ross S, Williams JG. 2007. Dictyostelium Myb transcription factors function at culmination as activators of ancillary stalk differentiation. *Eukaryot Cell* **6**: 568-570.

Wang Y, Senoo H, Sesaki H, Iijima M. 2013. Rho GTPases orient directional sensing in chemotaxis. *Proc Natl Acad Sci U S A* **110**: E4723-4732.

Warner N, Rutherford CL. 2000. Purification and cloning of TF2: A novel protein that binds a regulatory site of the gp2 promoter in Dictyostelium. *Arch Biochem Biophys* **373**: 462-470.

Wen X, Lei YP, Zhou YL, Okamoto CT, Snead ML, Paine ML. 2005. Structural organization and cellular localization of tuftelin-interacting protein 11 (TFIP11). *Cellular and molecular life sciences : CMLS* **62**: 1038-1046.

Wilsker D, Probst L, Wain HM, Maltais L, Tucker PW, Moran E. 2005. Nomenclature of the ARID family of DNA-binding proteins. *Genomics* **86**: 242-251.

Winckler T, Iranfar N, Beck P, Jennes I, Siol O, Baik U, Loomis WF, Dingermann T. 2004. CbfA, the C-module DNA-binding factor, plays an essential role in the initiation of Dictyostelium discoideum development. *Eukaryot Cell* **3**: 1349-1358.

Winter E. 2012. The Sum1/Ndt80 transcriptional switch and commitment to meiosis in Saccharomyces cerevisiae. *Microbiol Mol Biol Rev* **76**: 1-15.

Wu CL, Zukerberg LR, Ngwu C, Harlow E, Lees JA. 1995. In vivo association of E2F and DP family proteins. *Molecular and cellular biology* **15**: 2536-2546.

Wu L, Wu H, Ma L, Sangiorgi F, Wu N, Bell JR, Lyons GE, Maxson R. 1997. Miz1, a novel zinc finger transcription factor that interacts with Msx2 and enhances its affinity for DNA. *Mechanisms of development* **65**: 3-17.

Yamada Y, Cassidy A, Schaap P. 2018. The transcription factor Spores Absent A is a PKA dependent inducer of Dictyostelium sporulation. *Scientific reports* **8**: 6643.

Yamada Y, Kay RR, Bloomfield G, Ross S, Ivens A, Williams JG. 2010. A new *Dictyostelium* prestalk cell sub-type. *Dev Biol* **339**: 390-397.

Yamada Y, Wang HY, Fukuzawa M, Barton GJ, Williams JG. 2008. A new family of transcription factors. *Development* **135**: 3093-3101.

Yamamoto M, Ko LJ, Leonard MW, Beug H, Orkin SH, Engel JD. 1990. Activity and tissue-specific expression of the transcription factor NF-E1 multigene family. *Genes & development* **4**: 1650-1662.

Yamane J, Kubo A, Nakayama K, Yuba-Kubo A, Katsuno T, Tsukita S, Tsukita S. 2007. Functional involvement of TMF/ARA160 in Rab6-dependent retrograde membrane traffic. *Experimental cell research* **313**: 3472-3485.

Yu B, Fey P, Kestin-Pilcher KE, Fedorov A, Prakash A, Chisholm RL, Wu JY. 2011. Spliceosomal genes in the D. discoideum genome: a comparison with those in H. sapiens, D. melanogaster, A. thaliana and S. cerevisiae. *Protein Cell* **2**: 395-409.

Zheng N, Fraenkel E, Pabo CO, Pavletich NP. 1999. Structural basis of DNA recognition by the heterodimeric cell cycle transcription factor E2F-DP. *Genes & development* **13**: 666-674.

Zhukovskaya NV, Fukuzawa M, Tsujioka M, Jermyn KA, Kawata T, Abe T, Zvelebil M, Williams JG. 2004. Dd-STATb, a Dictyostelium STAT protein with a highly aberrant SH2 domain, functions as a regulator of gene expression during growth and early development. *Development* **131**: 447-458.
